# Fly-scan-oriented motion analyses and upgraded beamline integration architecture for the High-Dynamic Double-Crystal Monochromator at Sirius/LNLS

**DOI:** 10.1107/S1600577522010724

**Published:** 2023-01-01

**Authors:** Renan Ramalho Geraldes, Sergio Augusto Lordano Luiz, João Leandro de Brito Neto, Telles René Silva Soares, Ricardo Donizeth dos Reis, Guilherme A. Calligaris, Gert Witvoet, J. P. M. B. Vermeulen

**Affiliations:** a Brazilian Synchrotron Light Laboratory (LNLS), Brazilian Center for Research in Energy and Materials (CNPEM), Rua Giuseppe Máximo Scolfaro, 10000 Campinas, São Paulo, Brazil; b Eindhoven University of Technology, Department of Mechanical Engineering, Control Systems Technology Group, De Zaale, 5612 AJ Eindhoven, The Netherlands; c TNO, Optomechatronics Department, Stieltjesweg 1, 2628 CK Delft, The Netherlands; Advanced Photon Source, USA

**Keywords:** vertical double-crystal monochromator, synchrotron instrumentation, high-performance mechatronics, continuous energy scanning, beamline integration

## Abstract

The High-Dynamic Double-Crystal Monochromator (HD-DCM) is a mechatronic system with unique control-based architecture and deep paradigm changes as compared with traditional beamline monochromators. Aiming at unprecedented inter-crystal positioning stability in vertical-bounce double-crystal monochromators (DCMs) of the order of 10 nrad RMS (1 Hz to 2.5 kHz), and not only in fixed-energy but also in fly-scan operation, it has been developed according to a ‘first-time right’ predictive design approach for hard X-ray beamlines at Sirius, the fourth-generation light source at the Brazilian Synchrotron Light Laboratory (LNLS/CNPEM). This work explores some of the challenges that emerge with this new technology and presents the latest commissioning results that demonstrate the unparallel performances of the HD-DCM at the undulator-based EMA (Extreme Methods of Analysis) beamline at Sirius. With the enabled fast spectroscopy fly-scan possibilities, a new energy-tuning evaluation method, based on wave-propagation simulations, becomes part of a motion-oriented analysis that is carried out to derive the multi-axis non-linear positioning problem, covering not only energy selection and fixed exit in the HD-DCM but also the emission spectrum of an adjustable-phase undulator (APU). The HD-DCM control scheme and its flexible operation modes are described in detail as well. Furthermore, a new integration topology between the HD-DCM and EMA’s APU, coming already close to ultimate motion levels, is described and validated.

## Abstract 2

The High-Dynamic Double-Crystal Monochromator that has been developed for the fourth-generation light source Sirius/LNLS is explored, with its unprecedented inter-crystal stability performance around 10 nrad RMS both at fixed-energy and continuous fly-scan. Given the new paradigm and the unique enabling technology of its high-performance mechatronic system, the engineering and operational aspects for the next-level beamline experimental possibilities with a double-crystal monochromator are comprehensively discussed. Fly-scan spectroscopy scientific commissioning results are also presented.

## 1. Introduction

At synchrotron light sources, hard X-ray monochromatic beams are often provided by monochromators that are implemented as a non-dispersive parallel arrangement of two identical monocrystalline structures. Constructively, this arrangement can be implemented in two basic ways, namely: (1) a single crystal with a groove, a design concept known as channel-cut; and (2) two independent crystals, generally referred to as a double-crystal monochromator (DCM). This work is focused on DCMs, whose key conceptual advantage over channel-cuts is the ability to keep a fixed-exit beam independently of the rotation of the crystals for energy selection, which can be important for beamline alignment purposes and relative motion of the beam with respect to the sample under analysis.

The first DCMs were developed in the late 1970s and early 1980s (Hastings, 1977 ▸; Lemonnier *et al.*, 1978 ▸; Golovchenko *et al.*, 1981 ▸; Hussain *et al.*, 1982 ▸; Cowan *et al.*, 1983 ▸), since when incremental upgrades have been gradually implemented to adapt to ever more stringent requirements over time (Kelly *et al.*, 2013 ▸; Kristiansen *et al.*, 2015 ▸; Waterstradt *et al.*, 2018 ▸; Baker *et al.*, 2018 ▸; Dolbnya *et al.*, 2018 ▸; Fan *et al.*, 2020 ▸). This evolutionary approach has generally persisted even after the workshop on X-ray DCMs, held in 2014 by the European Synchrotron Radiation Facility in Grenoble, France (http://www.esrf.eu/home/events/conferences/2014/double-crystal-monochromator-workshop.html), in which it was recognized by a great number of worldwide experts in the field that no existing DCM was able to meet some of the requirements of the new-generation beamlines. Indeed, starting in the 2010s, the new fourth-generation storage rings push the X-ray brightness and coherence fraction to unprecedented levels, opening unique science opportunities in terms of temporal and spatial resolutions (Eriksson *et al.*, 2014 ▸; Hettel, 2014 ▸), but require that the beamline instrumentation performs accordingly. Still, after so many years of development, the main recurrent challenge in the DCMs is found to be keeping the inter-crystal parallelism, so that variations in flux and beam position are kept within acceptable levels, while handling multiple moving axes, high power loads, radiation and in-vacuum operation.

Aiming at improved fixed-energy stability and unlocking high-performance fly-scan perspectives with the inter-crystal parallelism target of 10 nrad RMS (1 Hz to 2.5 kHz), the Brazilian Synchrotron Light Laboratory (LNLS/CNPEM) has developed the High-Dynamic Double-Crystal Monochromator (HD-DCM) for the fourth-generation light source Sirius (Liu *et al.*, 2021 ▸), the first DCM to implement a high-performance control-based isolated mechatronic architecture. Herein, *fixed-energy* or *stand-still* is understood as all motion axes in position during data acquisition, which can also be related to traditional *step-scan* operation, whereas *fly-scan* refers to continuous synchronous motion of axes with simultaneous data acquisition. The first HD-DCM unit has been operational at the MANACÁ (MAcromolecular micro and NAno CrystAllography) beamline since 2020, mostly working on fixed-energy experiments, whereas the second unit at the EMA (Extreme Methods of Analysis) beamline has been operational since 2021, where the scanning perspectives started to be explored.

To advocate in favor of its powerful technology and the applied development methodologies, and allay insecurities in the community, a lot has been shared over the years at topical conferences: the basic conceptual design, mechatronic principles and thermal management were described by Geraldes *et al.* (2017*a* ▸,*b* ▸) and Saveri Silva *et al.* (2017 ▸); the first results of in-air validation of the core, together with system identification and control techniques in the prototyping hardware, were shown by Caliari *et al.* (2018 ▸) and Moreno *et al.* (2018 ▸); the offline performance of the full in-vacuum cryocooled system, including energy fly-scan, was demonstrated by Geraldes *et al.* (2018) ▸; the dynamic modeling work, updated control design and NI LabVIEW FPGA implementation in the final NI’s CompactRIO (cRIO) were addressed by Geraldes *et al.* (2020 ▸), Caliari *et al.* (2020 ▸) and Moraes *et al.* (2020 ▸); calibration and commissioning procedures, together with the first experimental results with beam, were shown by Geraldes *et al.* (2021*a* ▸); and, finally, integration aspects with undulator sources started to be discussed by Geraldes *et al.* (2021*b* ▸). More recently, the detailed mechanical design and mechatronic architecture of the HD-DCM has been thoroughly described by Geraldes *et al.* (2022*a* ▸,*b* ▸); whereas, together with updated commissioning results, a didactic discussion on the fundamental conceptual differences between the HD-DCM and standard DCM mechanical architectures has been provided by Geraldes *et al.* (2022*c* ▸).

This work mainly focuses on detailing and experimentally validating the positioning problem formulations for both the HD-DCM and its current adjustable-phase undulator (APU) source from an energy fly-scan (or spectroscopy) perspective. Indeed, although basic geometrical and physical equations can be found elsewhere (Hidas *et al.*, 2022 ▸), to the best of the authors’ knowledge a mechatronic approach concerning ranges, velocities, acceleration levels, forces and torques has not been covered in the literature yet — perhaps due to step-based actuation technologies and dominant fly-scan limitations related to motion errors in standard DCMs. With the HD-DCM, however, this understanding is essential to realize the full potential of the instrument, allowing for high-stability fixed-exit spectroscopy experiments to reach the order of 1 s, *i.e.* a time reduction of up to two or three orders of magnitude with respect to standard step-scans — which might drastically increase experimental throughput and/or even create new scientific perspectives. To that end, a partly tutorial approach is chosen here, such that the well known geometrical and physical properties within a DCM scope can be revisited from the necessary mechatronic approach, from where practical limits, tuning tolerances, calibration strategies and advanced beamline integration needs can be derived.

This paper is organized as follows. Firstly, in Section 2, the energy selection and fixed-exit concepts of a DCM, both in nominal geometry and including crystal miscut, are used to derive the non-linear characteristics of a DCM and its implications in fly-scans. Then, in Section 3, the relevant aspects of APUs regarding their integration with the HD-DCM, particularly focusing on tuning tolerances and fly-scan positioning demands, are developed. Next, Section 4 describes the generic and flexible beamline integration architecture developed for the HD-DCM, as well as its operation modes, and the latest communication topology with its APU source, which finally enabled practical high-performance fly-scan spectroscopy. After that, Section 5 summarizes some experimental results at EMA, including: (1) stand-still inter-crystal stability measurements via rocking-curve edges and knife-edge; (2) fly-scan motion performance evaluation according to the new integration architecture between the HD-DCM and APU; and (3) a long-term scientific high-pressure commissioning experiment via step and fly-scan spectroscopy. Conclusions and further considerations are given in Section 6. Also, a list of abbreviations is provided for general reference in Table 1 ▸.

## 2. DCM positioning problem formulation

**Figure 1:**
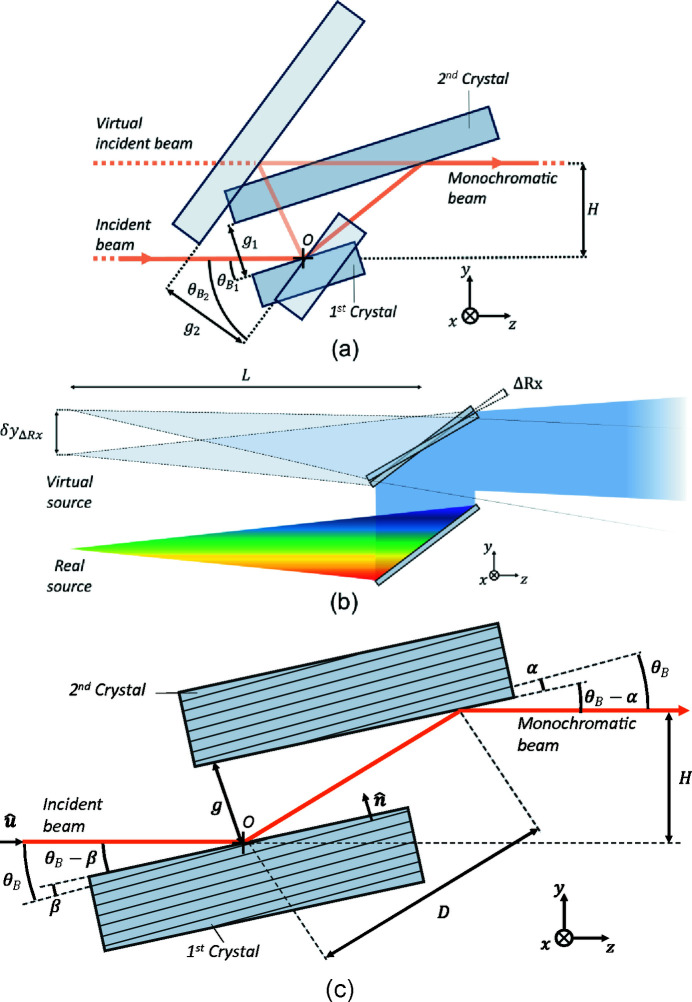
(*a*) DCM geometry according to the fixed beam impact point at the rotation axis *O*, showing Bragg angles 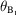 and 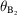 and the corresponding gaps *g*_1_ and *g*_2_, keeping constant offset *H* between the monochromatic and incident beams. (*b*) Effect of DCM inter-crystal parallelism variation Δ*Rx* in the position of the virtual source δ*y*_Δ*Rx*_. (*c*) DCM geometry including α and β miscut angles in asymmetric crystals, where 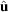 represents the unit vector of the incident beam, 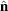 the unit vector normal to the diffraction planes, which is made parallel to the gap *g* that defines the offset *H*, and *D* is the distance traveled by the beam between crystals [adapted from Sterbinsky & Heald (2021 ▸)].

The main geometrical concepts of a DCM are illustrated in Fig. 1 ▸, which is thoroughly explored in the following subsections to first describe a nominal geometry, then elaborate on crystal asymmetry effects, and finally derive the main time-dependent fly-scan aspects, with highly non-linear motion characteristics that pose challenging requirements in terms of actuation, metrology, control and integration.

### 2.1. Nominal case

The energy selection in a DCM results from Bragg’s law of diffraction (Ashcroft, 2003 ▸), which describes that only photons of a given fundamental energy *E* and its harmonics are diffracted for a given incidence Bragg angle θ_B_, *i.e.*


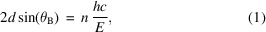

where *d* is the crystal lattice parameter, also known as *d*-spacing, *n* a positive integer that defines the diffraction order, *h* is Planck’s constant and *c* is the speed of light. Thus, different energies can be selected by adjusting θ_B_. Yet, in reality, the Bragg angle and the energy are not discrete values, but narrow distributions defined by an intrinsic crystallographic property, the so-called *Darwin width* (Koningsberger & Prins, 1988 ▸), such that the monochromatic beam results from a convolution of the diffraction in both crystals.

Hence, for two crystals with identical *d*, an ideal monochromatic beam, *i.e.* parallel to the incoming beam and with fixed exit, is nominally achieved by having parallel crystals and adjusting the gap *g* between them, such that a constant offset *H* is obtained according to 

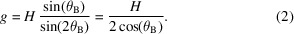

This is illustrated for two arbitrary angles in the side-view schematic of Fig. 1 ▸(*a*), which also follows the specific geometrical architecture chosen for the HD-DCM, namely: with the center of rotation of the crystals set on the surface of the first crystal; and with a long second crystal to comply with the beam walk for the different energies — *i.e.* preventing a complementary motion stage that otherwise would be needed to longitudinally move a short second crystal according to the different downstream positions of the beam after the diffraction in the first crystal.

Partially differentiating (1) with respect to *E* and *d* individually, and applying simple manipulation, it can be shown that 

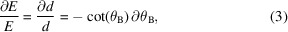

from where a few aspects can be highlighted. Firstly, it can be seen that the sensitivity to variations in θ_B_ in the energy selection starts from zero at the upper limit of θ_B_ = π/2 and tends to infinity as the Bragg angle moves towards θ_B_ = 0. Secondly, a given percentage of change in *d*-spacing is one-to-one related to the percentage of change in energy. Thus, if different *d*-spacings are found in the two crystals, an ideal energy matching for maximum flux would be related to slightly different θ_B_ in the crystals, such that the monochromatic beam would no longer be exactly parallel to the incoming beam, and the magnitude of the deviation would be variable over the operational energy range.

In practice, even for nominally identical crystals, *d*-spacing variations may result from crystal imperfections or clamping distortions, but they are most noticeably related to thermal effects. Indeed, *d*-spacing variations due to thermal expansion, together with local curvature in the crystal lattice, are well known issues in DCMs (and other types of monochromators) due to the local heat loads deposited in the first crystal, which may reach hundreds of W mm^−2^ (Zhang *et al.*, 2013 ▸). This effect tends to be highly non-linear in energy, since it may depend on: (1) the power variation as a function of energy for undulators; (2) the particular crystal heat extraction capacity; and (3) the power load distribution over the beam footprint *b*, which can be written as a function of θ_B_ and the beam size *a* as 

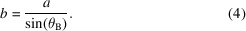

Clearly, this is in contrast with the concept of an ideal DCM, requiring some kind of compromise or compensation strategy regarding flux, beam parallelism and/or beam position at a given point of interest, as elaborated next.

Figure 1 ▸(*b*) illustrates how a small angle Δ*Rx* between crystals is related to a shift δ*y*_Δ*Rx*_ of the virtual source according to 

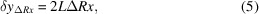

where *L* is the distance between the DCM and the source. Then, considering that variations of the virtual source are often proportionally related to shifts of the beam at the sample through the beamline optics, a common requirement is having them small compared with the source size. With X-ray source sizes of about 5 µm and *L* commonly of the order of 30 m for modern beamlines, a typical budget of 10% pushes Δ*Rx* to the range of 10 nrad only. This immediately reveals the stringent dynamical angular stability requirements for state-of-the-art DCMs, as well as suggests that within an experiment there would be virtually no margin for intentional variations of Δ*Rx*, for thermal effects compensation, for instance.

Yet, recalling (2), it can be seen that displacements in the virtual source related to Δ*Rx* may be at least partly compensated by energy-dependent beam offset corrections via gap adjustments. Again using partial derivatives, δ*y*_*H*_ can be written as 

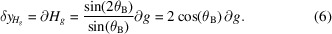

Moreover, although not practically useful for intentional offset compensation because of its correlation with energy, it should be noted that the offset is also sensitive to variations in θ_B_ according to 

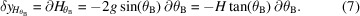

**Figure 2:**
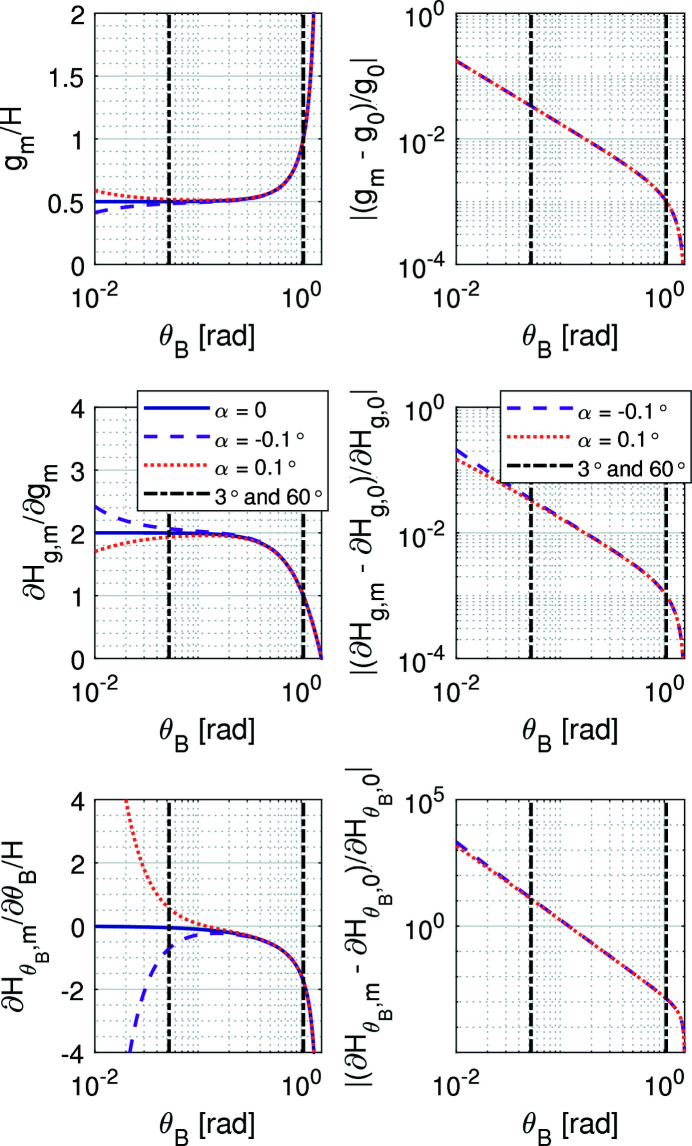
Gap *g* and offset *H* dependences on the Bragg angle θ_B_ for the DCM geometry of Fig. 1 ▸, including representative miscut manufacturing limitations, *i.e.* crystals with small asymmetric cut angles α. The quantities of interest are shown on the left and the relative ratio with respect to the nominal case on the right. The θ_B_ axis is shown in logarithmic scale to highlight the contribution of the miscut at lower angles (higher energies). The HD-DCM angular limits are represented by the vertical dash-dotted lines at 3° and 60°.

Indeed, in addition to compensations with 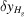 , these equations can be directly used for motion error specifications [see also Geraldes *et al.* (2022*a* ▸)]. It can be seen (refer also to Fig. 2 ▸ in Section 2.2) that at low energies (high angles) the offset is sensitive to changes in θ_B_ but quite insensitive to the gap *g*, and vice-versa. This is unfortunate, since the higher chances of corrections related to thermal effects occur precisely at low energies, where higher power densities occur due to smaller footprints [see (4)]. Thus, acceptable boundaries may need to be identified for each experiment individually. Ultimately, the target would be to have the effective virtual source variation δ*y* = 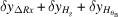 in an experiment within a fraction of the source size, *i.e.* typically about 0.5 µm, or the corresponding behavior at the sample position.

The angular boundaries for Δ*Rx* around an ideal energy tuning, which might be already out or perfect parallelism due to *d*-spacing variations, can be derived as a fraction of the angular bandwidth Δθ_DW_ of the Darwin width of the crystals. This can be used, for example, to evaluate acceptable flux losses in trying to keep the incoming and outgoing beam parallel despite thermal effects. It turns out that Δθ_DW_ can be described to a good approximation by a ‘rearrangement’ of (3), 

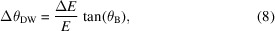

where Δ*E*/*E* becomes the intrinsic energy resolution of the crystal, a constant dimensionless physical quantity that is typically in the range between 10^−3^ and 10^−5^. With typical orientations of silicon crystals, such as Si(111) and Si(311), which are used in the HD-DCM, Δθ_DW_ varies from hundreds of µrad at large θ_B_ to sub-µrad at small θ_B_ (see also Section 5.2.1). As a side note, detuning of crystals also often finds practical use in harmonic rejection strategies.

### 2.2. Asymmetric crystals

One last factual aspect to be considered is the inevitable existence of deviations between the actual crystal surface and its diffraction planes. Although many times asymmetric cut crystals reaching several degrees are intentionally designed for different purposes, including beam compression or expansion [see, for example, Hastings (1977 ▸)], here ideally symmetric-cut crystals with only manufacturing miscut limitations are considered. This is depicted in Fig. 1 ▸(*c*), which is adapted from the discussion recently carried out by Sterbinsky & Heald (2021 ▸) on how miscuts affect the gap in DCMs, where it can be seen that, for parallel diffraction planes in the first and second crystals, the distance between their surfaces varies over the propagation of the beam as a function of the small miscut angles β and α, respectively.

Considering here the gap value to be taken perpendicularly to the diffraction plane of the crystals and aligned with the rotation axis, a derivation equivalent to that described by Sterbinsky & Heald (2021 ▸) shows that (2) becomes 

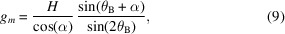

from where it can be seen that, in addition to the gain 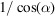 , which is very close to unity for small α, the larger deviations from (2) occur for small θ_B_. Then, updating (6) and (7) yields 

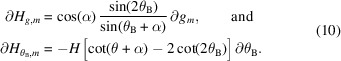

One may notice that β does not appear explicitly in any of these equations. This is because no beam walk is assumed in the first crystal. In practice, with imperfect alignment of the crystal surface together with the incidence beam on the axis of rotation, parasitic beam walk will occur, resulting in some contribution from β to the gap. Nonetheless, in this geometry, this contribution will be negligible compared with that of α.

Figure 2 ▸ shows plots comparing these quantities for α = 0, *i.e.* the nominal case with no miscut, and two miscut cases, with α = ±0.1° to represent reasonable manufacturing limitations. The angular range from 0.01 rad ≃ 0.57° to π/2 rad = 90° is shown in logarithmic scale for clarity, and the angular limits of the HD-DCM are represented by the vertical dash-dotted lines at 3° and 60° for reference. The dimensionless quantities are shown on the left side, whereas the relative ratio with respect to the nominal case on the right. In the upper plots, it can be seen that the gap no longer tends to an asymptotic value as θ_B_ becomes smaller for higher energies. Similarly, the remaining plots demonstrate the increased sensitivity in the offset position to both the gap and θ_B_, the latter in particular increasing by several orders of magnitude, when miscuts are considered.

This indicates that in reality the gap motion range may need to be larger by a few percent than nominally expected, that sensitivities at low angles may vary by more than one order of magnitude, that calibrations for fixed exit may require more than the simpler trigonometric relation of (2) [see also Sterbinsky & Heald (2021 ▸)], and that the required velocities and accelerations related to the fly scan are in practice different from nominal ones.

### 2.3. Fly-scans

To conclude, the previous equations can be further discussed in terms of their implications in fly-scan spectroscopy. Firstly, for θ_B_, *E* from (1) can be substituted into (3), and the result taken with respect to time, such that 

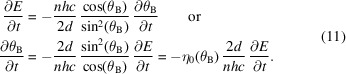

Then, for the gap, (9) can be derived for a constant offset with respect to time, yielding 

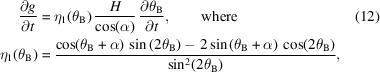

which, in terms of the energy rate, using (11), becomes 

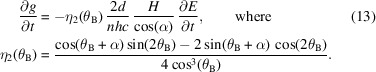

Naturally, here θ_B_ in the trigonometric arguments are actually related to *E* via (1), but replacing it with the inverse trigonometric function does not add to clarity.

**Figure 3:**
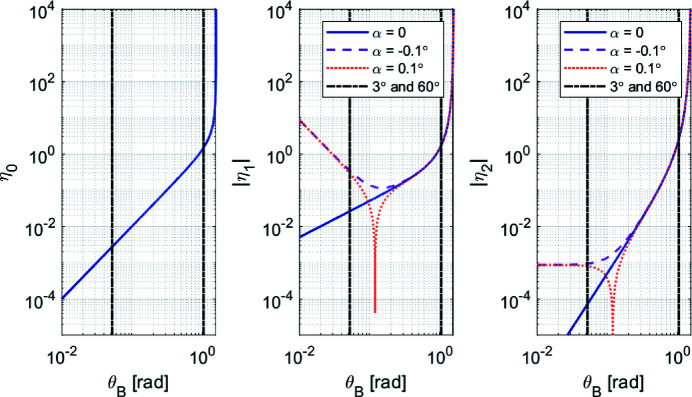
Trigonometric functions related to the variation of: the Bragg angle θ_B_ with respect to energy *E*, as η_0_; and the gap *g* with respect to θ_B_ and *E*, as η_1_ and η_2_ (shown in absolute values for readability in the logarithmic scale), respectively. They follow (11), (12) and (13) for the DCM geometry of Fig. 1 ▸, including representative miscut manufacturing limitations, *i.e.* crystals with small asymmetric cut angles α. The HD-DCM angular limits are represented by the vertical dash-dotted lines at 3° and 60°.

The functions η_0_, η_1_ and η_2_ are introduced in (11), (12) and (13) to simplify the notation of these equations by capturing the complex trigonometric dependences on θ_B_. Indeed, from Fig. 3 ▸, the strong non-linear relationships in following the desired energy variation rates in fly-scans become evident. From η_0_, the exponential increase in the required Bragg velocity for higher angles (lower energies) is clearly visible, changing by nearly three orders of magnitude within the operational range of the HD-DCM. From η_1_ and η_2_, although with different slopes, similar exponential behavior occurs for the gap with the nominal geometry with α = 0. With miscuts, however, deviations occur in the low-angle (high-energy) range, as also seen in Fig. 2 ▸. In particular, because η_2_ — representing the variation of *g* with respect to *E* — is the product of η_0_ and η_1_, it actually asymptotically converges to a small constant value, either for positive or negative α. Hence, differences of three to four orders of magnitude occur for the gap velocity for a given energy variation rate within the operational range of the HD-DCM.

For a control-based instrument like the HD-DCM, these aspects place demanding specifications on metrology and acquisition hardware, since very high resolution and low noise are required for the lower angular (higher energy) range, whereas high rates are necessary at the opposite limit. For example, while the angular resolution in the Bragg angle quadrature encoder is 50 nrad (or about 3 µ°) for high angular resolution and small control errors, from (11) it can be found that, for an energy scan of 1 keV s^−1^, the Si(111) crystal angular speed requirements would be around 0.1° s^−1^ at the high energy range and as much as 40° s^−1^ at the low energy limit. In the latter case, the counting rates would have to be higher than the current electronics capacity of 10 MHz. Similarly for the gap, with a resolution of 0.1 nm from the quadrature laser interferometers for the nanometre-level control performance, an equivalent energy rate scan speed with Si(111) crystals without a miscut would translate via (13) to about 0.8 µm s^−1^ and 20 mm s^−1^ at the high and low energy limits, respectively. In the latter case, counting rates would need to reach 200 MHz.

Moreover, reaching such high speeds in a reasonable traveling range may be associated with significant acceleration levels, since the main rotation mass moment of inertia and the gap stage mass are close to, respectively, 14 kg m^2^ and 6 kg [see also Geraldes *et al.* (2022*b* ▸)]. Indeed, taking the derivatives of (11), (12) and (13) with respect to time, the acceleration levels might span over even higher ranges than those of velocity, stressing the demands on the actuators, amplifiers and converters, again with very high resolution and low noise for the lower angular range, but higher forces and torques at the opposite limit. Furthermore, even considering the control-based and disturbance-oriented design of the HD-DCM, the higher speeds and accelerations at higher angles inevitably increase mechanical and setpoint-related disturbances.

The performance of the HD-DCM under these challenges and the ongoing research are discussed via results in Section 5, but, before that, the following section elaborates a corresponding analysis for an APU source, showing how it impacts energy fly-scans with the HD-DCM.

## 3. Undulator properties and positioning analyses

So far, Sirius beamlines have been designed with two types of sources; namely, bending magnets, from the 3.2 T permanent-magnet dipoles that are part of its five-bend achromat lattice (5BA) (Liu *et al.*, 2021 ▸), and undulators. The first provides a continuous and broad-band spectrum (with critical energy at 19 keV), that can be used in a number of beamlines, for different characterization methods, and is transparent with respect to a DCM operation [aside from power density variations due to the footprint dependence on θ_B_, as seen in (4)]. The latter, on the other hand, has an adjustable photon emission spectrum, justifying the specification of a particular device for each beamline individually, depending on the scientific research area, and requiring energy tuning with the monochromator. Therefore, key undulator characteristics and positioning tolerances for energy fly-scans with the HD-DCM at Sirius are investigated next. The analyses will be restricted to the case of APUs, which is the first type to be integrated with the HD-DCMs at Sirius beamlines, but, naturally, equivalent analyses can be made for different types of undulators.

### 3.1. Undulator emission properties

**Figure 4:**
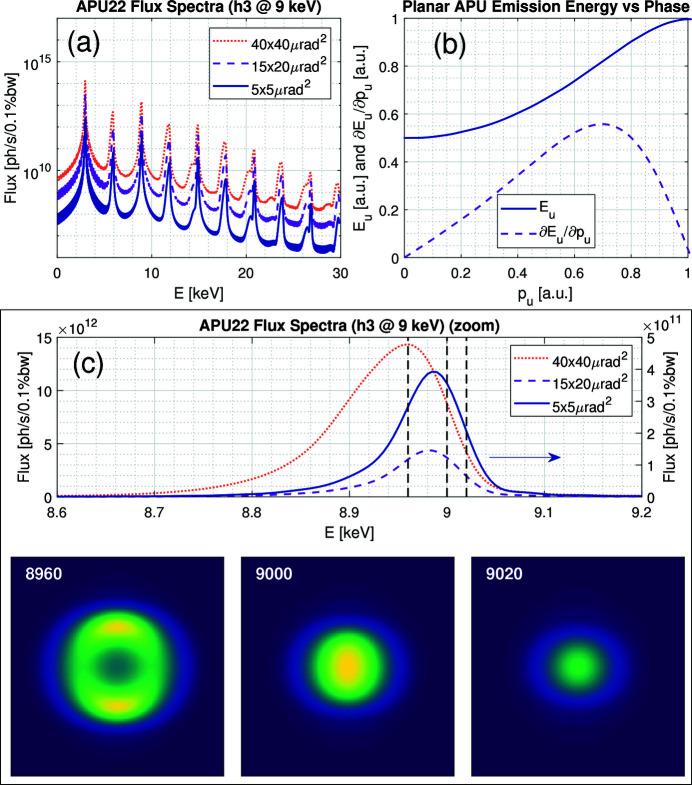
(*a*) Example of the emission flux spectra of the planar adjustable-phase undulators APU22 installed at EMA and MANACÁ at Sirius, with the third harmonic tuned at 9 keV. The first nine harmonics are shown for a given deflection parameter *K*_u_, and three different beamline angular acceptance windows (v × h) illustrate its effect in peak energy width and flux. (*b*) Arbitrarily scaled energy value *E*_u_ for a given emission harmonic *n*_u_ and its derivative with respect to phase, for a normalized undulator phase *p*_u_ in a planar APU, according to (14). (*c*) Top: zoomed spectral flux plot of the example emission of (*a*), highlighting the broadening of the peak and its shift to lower energies for larger angular acceptances; bottom: ‘slitless’ wave-propagation simulations showing the beam profile for perfectly monochromatic energies at and around the 9 keV resonance (black vertical dashed lines in the plot).

Indeed, as illustrated in Fig. 4 ▸(*a*), the flux in an undulator spectrum is characterized by relatively narrow emission bands, which are harmonics of a fundamental energy and depend on physical constants and parameters both of the storage ring and of the particular device. Then, by online adjustment of the so-called undulator deflection parameter *K*_u_ — that is a function of its magnetic period λ_u_ and magnetic field *B*_u_, which may have components in both axes transversal to the propagation of the electron beam — the energy spectrum and possibly the polarization of the photon beam can be selected accordingly by the user. The plot covers the first nine harmonics of the APU22 undulator that is currently installed at both EMA and MANACÁ beamlines, in an arbitrary configuration with the third harmonic tuned to 9 keV for a given *K*_u_. It clearly illustrates the gradual reduction in flux over several orders of magnitude as the harmonic order increases towards higher energies, and also that the peak energy width and the flux depend on the angular acceptance window at the beamline.

**Figure 5:**
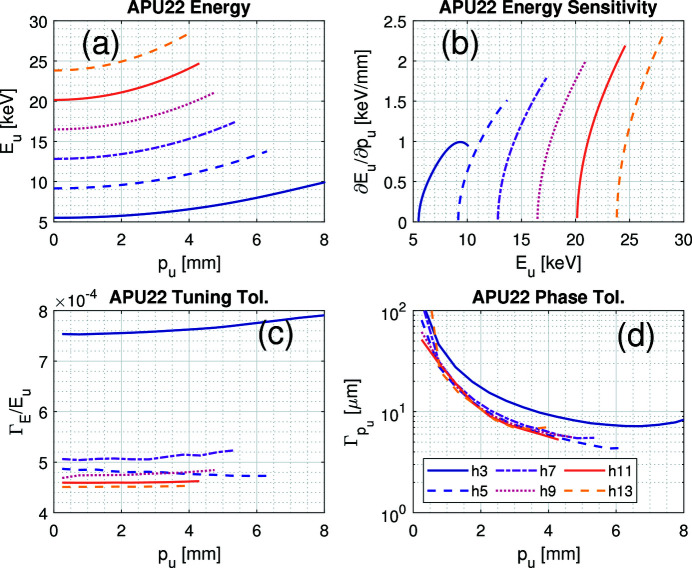
APU22 characteristics for the different harmonics in the energy range between 5.7 and *28* keV at the EMA and the MANACÁ beamlines at Sirius: (*a*) peak emission energy *E*_u_ as a function of the phase *p*_u_; (*b*) variation correlation between *E*_u_ and *p*_u_ as a function of *E*_u_; (*c*) energy tuning tolerance ratio as a function of *p*_u_; and (*d*) phase error tolerance 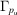 as a function of *p*_u_.

In APUs, *K*_u_ can be varied from a maximum value to virtually zero by relatively shifting one of its magnetic cassettes with respect to the other, defining a variable generally referred to as the undulator phase *p*_u_, which has a typical stroke of the order of 10 mm. Without diving into the development of the equations and into the physical parameters, as given in detail by Onuki & Elleaume (2002 ▸), it can be shown that an emission flux peak for a given energy *E*_u_ can be nominally written in terms of *p*_u_ as 

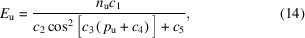

where *n*_u_ is the emission harmonic and *c*_*i*_, with *i* = 1,…5, are constants resulting from physical constants and parameters of the storage ring and of the device. Thus, the peak emission energy dependence on *p*_u_ for such an APU has a generic shape as depicted for normalized phase values in Fig. 4 ▸(*b*), *i.e.* ranging from a minimum to a maximum value for each harmonic. In most cases, the harmonics overlap, meaning that the same energy may be reached in two or more harmonics/phases, only with different fluxes (see also Fig. 5 ▸ in Section 3.2). This is especially useful for energy scans realized around the overlapping regions, such that full scans can be executed without commanding the undulator over discontinuous trajectories, which would be actually impractical in fly-scans. In other cases, energy gaps may exist, meaning that peak emissions do not occur for a given energy range with the particular combination of parameters and would not be available to the user, being a design choice.

Consequently, for DCMs operating at undulator beamlines, θ_B_ must be tuned to *p*_u_ for the desired output energy. This aspect is illustrated in Fig. 4 ▸(*c*). Firstly, a zoomed spectral flux plot of the emission around the third harmonic at 9 keV is shown to illustrate in more details in linear scale (and with the smaller acceptance in the right axis for readability) the broadening of the peak and its shift towards lower energies as the angular acceptance is increased for higher fluxes. Then, wave-propagation simulations using Synchrotron Radiation Workshop [*SRW* (Chubar & Elleaume, 1998 ▸)] show the beam profile for perfectly monochromatic energies at and around the 9 keV resonances without any slit acceptance restrictions, from where it can be seen that, if the DCM and the APU are detuned beyond given energy-dependent margins, either the downstream flux is rapidly reduced to one side or the ideal Gaussian beam profile is distorted to ring-like structures to the other.

Therefore, for energy fly-scans at undulator beamlines the traditional stand-still tuning between θ_B_ and *p*_u_ becomes dependent on the motion capabilities of the undulator and synchronism as well. As done in the previous sections, (14) can also be derived as a function of time, resulting in 

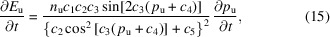

which can be used in (11) and (13) to find the required motion correlation between all the axes of interest for an energy scan with an APU and a DCM. This correlation between the variation of *E*_u_ and *p*_u_ is also qualitatively shown in Fig. 4 ▸(*b*), from where both the sensitivity in the emission spectrum due to motion errors in the undulator, for instance, as well as the effectiveness in realizing scans can be observed.

For a desired energy variation rate, large speeds would be required from the undulator motion at the phase limits. In addition, as for the DCM, linear energy scans require non-linear undulator trajectories, via an exotic function as compared with industrial standards, which may not be directly available for undulator controllers. So, it becomes clear that ideal and ‘simple’ user settings for constant energy scanning rates, in practice, must be translated according to highly non-linear functions, often tending either to zero, or infinity, as shown next for the APU22 at Sirius.

### 3.2. The APU22 source

The two operational units of the HD-DCM are at the EMA and the MANACÁ beamlines at Sirius, which currently operate in a commissioning configuration with an APU22 undulator source by Kyma: λ_u_ = 22 mm, length = 1.12 m and *K*_u,max_ = 1.44. By 2024, final configurations are expected, with two of the APU22 units in series for MANACÁ, and a new IVU18.5 (in-vacuum undulator) for EMA: λ_u_ = 18.5 mm, length = 2 m and *K*_u,max_ = 2.1.

Hence, in this early stage both beamlines are working with lower fluxes, not only because the storage ring current is still limited to 100 mA, while expected to reach 350 mA, but also because of the shorter undulator. Besides that, EMA is working with a more limited energy range, due to an existing energy gap between 3.7 and 5.7 keV, occurring between h1 and h3, *i.e.* the first and third harmonics (even harmonics do not provide adequate sources) of the APU22. Moreover, due to an initial perspective of short-time replacement (or upgrade) of the sources, only basic features have been originally specified for the APU22 Beckhoff’s control system. This has eventually posed integration limitations with respect to the HD-DCM in the early operation phase, motivating the development of alternative solutions, as discussed in the next sections.

Following the generic shape anticipated in Fig. 4 ▸(*b*), Fig. 5 ▸(*a*) shows the harmonic emission peaks between h3 and h13 as a function of phase, thus covering the energy range that has been explored so far at EMA and MANACÁ. Indeed, the useful range of h1 by the HD-DCM, *i.e.* between 2.3 and 3.7 keV, has a sharp flux reduction in this range (not shown) and would drastically suffer from attenuation from air and windows in the setups currently available at both beamlines. Higher energies, in turn, have prohibitively low fluxes with the APU22 and anyhow face reflectivity limitations from downstream mirrors. In the plot, the harmonics are truncated after an arbitrary overlap of 1 keV due to flux gains.

Then, Fig. 5 ▸(*b*) illustrates the variation correlation between the energy and the phase, but as a function of the energy for the various harmonics, instead of as a function of the phase as in Fig. 4 ▸(*b*). This can be directly used as a guide to evaluate how desired rates in keV s^−1^ are translated to mm s^−1^ in the undulator. For example, energy rates of 1 keV s^−1^ can be achieved with reasonable phase speeds in the range of 1 mm s^−1^ depending on the desired energy range, whereas it would require ‘infinite’ speed around energies close to *p*_u_ = 0. Before fast orbit feedback (FOFB) and feedforward systems, that should be capable of compensating possibly higher orbit distortions caused by the fast motion of the undulators, become available at Sirius’ storage ring, an upper limit velocity of 3 mm s^−1^ has been specified to the APU22. Yet, due to the current flux limitations, at EMA the APU22 has been operated in practice well below 1 mm s^−1^ in fly-scans, reaching only a few tens of eV s^−1^ in this initial stage. Hence, ultimate fly-scan spectroscopy velocities are expected to be achieved later, with the final sources and with the storage ring systems fully implemented.

Next, to define synchronism and motion performance requirements for the APU22, a tuning tolerance method using wave-propagation simulations like those in Fig. 4 ▸(*c*) has been developed. It consists of analyzing a finite number of energies in each harmonic, and, for each of them, mapping an energy tuning tolerance Γ_*E*_ corresponding to a variation of 10% of the full width at half-maximum (FWHM) of the beam with respect to the ideal ‘Gaussian-like’ profile. The ratio between the tolerances and the central (nominal) energies are shown in Fig. 5 ▸(*c*), in which it can be seen that there is a higher forgiveness, around 0.075%, in h3 (and even higher for h1, around 0.18%, not shown), but similar ratios for h5 to h13, around 0.045% to 0.05%. This is because h1 and h3 are related to lower energies in which the X-ray source size and divergence are larger due to diffraction limits (not shown), whereas beyond h5 these parameters reach asymptotic values. Occasional residual inaccuracies in the estimation of Γ_*E*_ seem insufficient to hinder any conclusions.

Finally, the dependence from Fig. 5 ▸(*b*) can be combined with the tolerances from Fig. 5 ▸(*c*) to determine phase tolerances 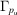 for the undulators. This is shown in Fig. 5 ▸(*d*), from where it can be seen that at the lower phase limit (<2 mm), related to higher velocities for given energy rates, the error tolerances exceed 10 µm, whereas only 4 to 7 µm are required as the most stringent values. At this point, it is worth noting that, using (3), the same energy tolerances from Fig. 5 ▸(*c*) can be taken to derive angular tolerances for the Bragg angle with respect to an ideal energy setpoint. It turns out that, within this energy range, these calculated tolerances remain above 30 and 60 µrad for Si(111) and Si(311), respectively, which are much larger than the typical motion errors observed in the HD-DCM, as shown in Section 5.3.1. Indeed, the obtained energy tolerances varied between 4 and 16 eV, whereas spectroscopy resolutions in this energy range generally aim at sub-eV targets, such that the Bragg angle performance should be truly at least one order of magnitude better than that.

Consequently, in practice the mapped tolerance budget does remain entirely for the undulator, but must include metrology accuracy, motion control errors, and delay effects, which might possibly lead to integration-related limitations beyond the performance of each instrument individually. This has been the case with the APU22 until very recently, as elaborated by Geraldes *et al.* (2021*b* ▸, 2022*c* ▸). And it highlights the need of a holistic approach in beamline instrumentation for increased performances and efficiency, as discussed in the following sections.

## 4. HD-DCM integration and operation schemes

Once the essential positioning characteristics for the HD-DCM and the APU22 have been explained — including higher-level motion parameters for fly-scan spectroscopy that are now for the first time enabled with the HD-DCM — this section summarizes how the HD-DCM can be effectively used at Sirius beamlines, including its integration schemes and operation modes. Sufficient details, also updated with respect to Geraldes *et al.* (2021*b* ▸), are given to clarify the several operational possibilities within more complex integration demands, leading to different hardware and software complexity requirements.

**Figure 6:**
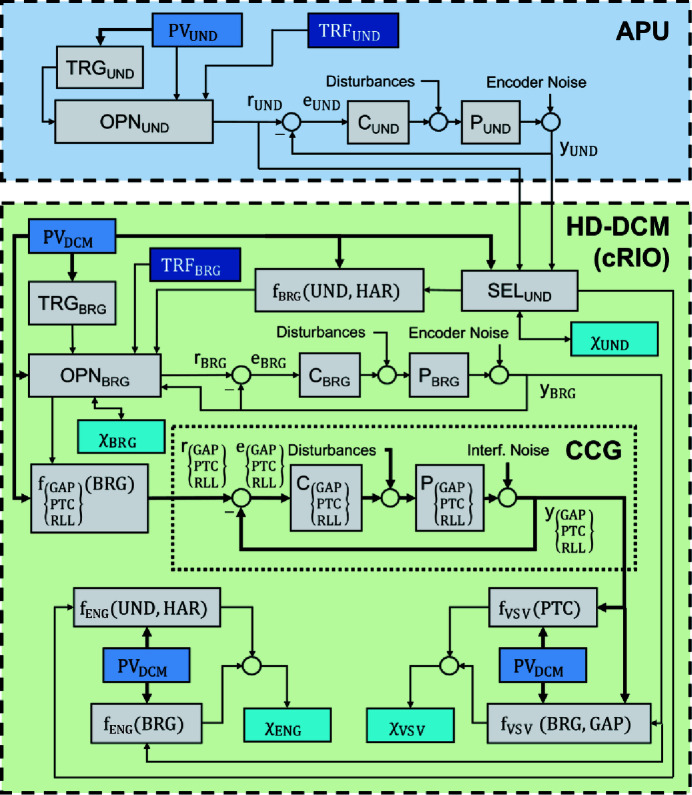
Simplified control implementation diagram for the integration of the HD-DCM running on NI’s cRIO, with four control loops for the Bragg angle (BRG) and crystal cage (CCG, with GAP, PTC and RLL) position control, and an APU source running on a third-party controller, with one control loop for the phase. Reference *r* and metrology *y* signals are represented, together with controllers *C*, plants *P*, and functions *f* for coordinated motion. As detailed in the text, motion setpoint updating depends on structures for input selection (SEL) and operation modes (OPN), including trajectory generation on demand (TRG), stored trajectory files (TRF) and motion estimators (χ_UND_ and χ_BRG_). Complementary real-time performance estimators can be computed for energy tuning (χ_ENG_) and virtual source vertical position (χ_VSV_). Integration with EPICS is represented by the process variables (PVs). Colors are given to differentiate the APU from the HD-DCM domains, and the control blocks from data blocks (related trajectories or PVs), and from the estimators.

The HD-DCM application within a flexible integration scheme with a generic APU source is illustrated in the simplified control implementation diagram of Fig. 6 ▸. Integration with the well known EPICS control system (https://epics-controls.org/), via the so-called process variables (PVs) for high-level user operation, is represented in the diagram by the abstracted multidimensional structures PV_UND_ and PV_DCM_, for variables related to the undulator and the HD-DCM, respectively.

The APU is represented by a generic single control loop UND for the phase (see Fig. 6 ▸), typically consisting of a servo-motor and a linear encoder in a third-party controller, with sample rates in the order of 1 kHz. Evidently, APUs with different embodiment or different types of undulators can be correspondingly adapted. The HD-DCM, in turn, can be defined by four main control loops (see Fig. 6 ▸), namely: Bragg (BRG), gap (GAP), pitch (PTC) and roll (RLL), running on NI’s CompactRIO (cRIO) hardware at 20 kHz sample rate [see also Geraldes *et al.* (2021*b* ▸, 2022*b* ▸)], as detailed next.

### 4.1. The HD-DCM control architecture

BRG is responsible for controlling the Bragg angle θ_B_ in the operational range from 3° to 60°, which is done via an in-vacuum direct-drive rotary stage with a rotary encoder, in a bandwidth of 20 Hz. Then, GAP, PTC and RLL, in turn, are part of the so-called *crystal cage* (CCG), responsible for controlling the position of the second crystal with respect to the first crystal with nanometre-level performance, which is done via voice-coil actuators and laser interferometers, in bandwidths between 150 and 250 Hz. GAP controls the parameter *g* from Section 2, whereas PTC and RLL, referring to the pitch and roll angles, control the inter-crystal parallelism. PTC is related to the crystals DW tuning and to vertical shifts in the virtual source via Δ*Rx* in (5), with the strict target of 10 nrad RMS (1 Hz to 2.5 kHz). RLL is related to lateral shifts in the virtual source, but with dependence on θ_B_ and much lower sensitivity than PTC, leading, for instance, to a positioning stability target of 90 nrad RMS. The complete set of specifications of the HD-DCM has been given by Geraldes *et al.* (2022*a* ▸).

Due to a careful mechatronic architecture, in practice the originally multiple-input–multiple-output (MIMO) system can be statically decoupled, so that the loops may be addressed independently and simpler single-input–single-output (SISO) controller design techniques can be used [see also Geraldes *et al.* (2022*b* ▸)]. Thus, in Fig. 6 ▸ the individual plants and controllers are represented as *P*_*i*_ and *C*_*i*_, respectively, where *i* refers to UND, BRG, GAP, PTC and RLL. Each loop follows a reference *r*_*i*_ and provides a measurement *y*_*i*_, being subject to actuator and plant disturbances, as well as measurement noise from sensors (encoders and laser interferometers). Measured error signals are defined as *e*_*i*_ = *r*_*i*_ − *y*_*i*_.

The reference signals for the CCG loops are defined as functions *f*_GAP_, *f*_PTC_ and *f*_RLL_ of θ_B_, which can follow nominal correlations, according to (2) or (9) for the gap, for instance, or result from beam-based calibrations [see Geraldes *et al.* (2021*a*) ▸], that can be updated by the user via PV_DCM_. Regarding the control-based architecture and fly-scan demands, it is useful to have these relations as continuous smooth functions, rather than arbitrary discrete look-up tables that might be used in step-scan applications, such that discontinuities are prevented and high-frequency content is minimized in the reference signals. So far, eighth-order polynomials have been used as a standard.

Furthermore, because the measurement signal *y*_BRG_ includes the disturbances in the BRG loop and electronic noise contamination that would deteriorate the individual performances of the CCG loops, it has been found that under normal conditions the control error *e*_BRG_ is sufficiently small that the reference signal *r*_BRG_ may be preferable as an indicator of θ_B_. Although perhaps not a usual control strategy in leading–following systems, it benefits from favorable tolerances in the HD-DCM and its reliability. Indeed, PTC and RLL have only (at most) a small and smooth dependence on BRG, resulting essentially from fine adjustment for parallelism and fixed-exit calibrations [see Geraldes *et al.* (2021*a*) ▸], with virtually no effect within small θ_B_ mismatching.

The GAP, in turn, as specified by Geraldes *et al.* (2022*a* ▸), can afford as much as 300 nm of mismatch in the worst-case scenario before its influence on the beam offset exceeds 10% of the beam size [see also (6) and (10)]. Then, using (2) — or, equivalently, (9) for miscuts — the derivative of the gap with respect to θ_B_ can be written as d*g*/dθ_B_ = 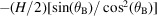 . Hence, with *H* = 18 mm and assuming an ideal gap motion, the corresponding BRG tracking mismatch, again for 10% of the beam sizes, should be bound to |*e*_BRG_| << 10 µrad in the 3° to 60° working range. As in the case of the energy tuning tolerances, this is one order of magnitude larger than the tracking errors that have been observed so far in fly-scans with the HD-DCM (see Section 5.3.1). This means that the loops can be connected only to a necessary extent, allowing for better individual performances, particularly of the PTC — which is the most sensitive degree of freedom, operating close to the specification boundaries.

A more elaborate alternative — that, however, has not proven practical until now — to prevent sensor noise, but still take the system response into account, consists of using an estimator χ_BRG_ of the closed-loop response via the complementary sensitivity function (*T*) of the BRG loop with χ_BRG_ = *T*_BRG_ *r*_BRG_. Whichever the case, the desired reference selection for the the CCG loops may be done in the operation mode block OPN_BRG_ via EPICS with PV_DCM_, which is also used to define the origin of the reference *r*_BRG_ itself.

Indeed, it is then the definition of how *r*_BRG_ is updated in the HD-DCM control loop that eventually defines how the instrument can be used at the beamline. In that sense, within the architecture in Fig. 6 ▸, three *operation modes* can be identified such that: (1) a trajectory is asynchronously calculated for *r*_BRG_ internally in the cRIO just before motion, being independent of other instruments; (2) a real-time conversion for *r*_BRG_ is made from a leading signal; and (3) a known trajectory for *r*_BRG_ is previously stored in a file in the cRIO and executed after a trigger for synchronism. As these modes are related to different experimental capabilities and implementation complexity concerning other instruments at the beamline, they are separately discussed in the following subsection [see also Geraldes *et al.* (2021*b*) ▸ for a preliminary discussion].

### 4.2. Operation modes

The first operation mode is the *stand-alone* mode, in which the HD-DCM can be operated by the user in a simple way at high level via PV_DCM_. In this case, a trajectory generator block TRG_BRG_ in cRIO uses user-defined parameters and default trajectory options to generate a suitable trajectory on demand and, then, triggers *r*_BRG_ to be updated accordingly at the 20 kHz sample rate. This is a basic mode for fixed-energy or step-scan experiments, and the undulator phase must be independently adjusted by the user via PV_UND_ for the appropriate energy tuning. Although not optimum in terms of timing, this can often be handled via standard step-scan beamline pieces of software. Moreover, this would also be a straightforward option for energy fly-scans at bending-magnet beamlines, in which the source spectrum is continuous.

Next, in the *follower* mode, *r*_BRG_ is updated in real-time as a function of a leading input signal, which might be any external reference, but here, naturally, consists of the undulator phase. Thus, *f*_BRG_ is defined as a function of the undulator phase and the desired harmonic (HAR), which is selected by the user as part of PV_DCM_. It can be nominally defined via (1) and (14), but, in practice, due to the relatively small tuning tolerances (see Section 3.2), requires beam-based calibration, as discussed by Geraldes *et al.* (2021*a*). Hence, by controlling the undulator alone via PV_UND_ with reasonable motion setpoints, the user should be able to obtain a well tuned fixed-exit monochromatic beam over the whole energy range, allowing the fly-scan spectroscopy potential to be explored at undulator beamlines. Integrating the HD-DCM with different types of undulators would basically require only adapting *f*_BRG_ to different parameters.

Still about the *follower* mode — in a similar fashion to the reference choice of the CCG loops — the phase signal can be also considered from three options that are managed via the selection block SEL_UND_, according to user settings in EPICS as part of PV_DCM_. They are: the measurement signal *y*_UND_; the reference *r*_UND_ from the undulator controller; or a position estimator χ_UND_ of the closed-loop response via the complementary sensitivity function with χ_UND_ = *T*_UND_ *r*_UND_ (as mentioned for the BRG loop). In the first, which was the original option available for the APU22, the encoder signal *y*_UND_ is directly derived to the cRIO — with the reading rates typically in the order of MHz for a quadrature signal, for example, being then internally downsampled to 20 kHz. However, as in the BRG case and the CCG loops, this signal includes disturbances from the UND loop and electronic noise that are propagated to *r*_BRG_. Thus, some filtering inside SEL_UND_ was found to be essential already at the early implementation phase. Nevertheless, as described by Geraldes *et al.* (2021*a* ▸, 2022*c*) ▸, even with the additional filters the performance degradation to the the HD-DCM loops ended up being unacceptably large for the desired fly-scan spectroscopy quality (with pitch stability exceeding 30 nrad RMS and correspondingly large Bragg control errors, for instance), such that further alternatives needed to be developed, as explained below.

Indeed, the two remaining options for the *follower* mode are based on the reference signal as the undulator phase indicator. Here, again connecting the loops only to the necessary extent, the tuning tolerances in the range of a few micrometres between the HD-DCM and the APU [see Fig. 5 ▸(*d*)] can be explored to leave out disturbances and noise from the undulator system, which is typically only a micrometre-level-performance machine. Naturally, both cases require a minimum level of reliability/repeatability in the undulator, which must be proven by each machine, and is demonstrated for the APU22 in Section 5.3. With *r*_UND_, the input may come via a digital signal from the undulator controller, with the advantage of being a noiseless signal, but numerical representation limitations and lower control rates may still exist, requiring additional filtering or interpolation functionalities in SEL_UND_ to prevent significant setpoint discontinuities in *r*_BRG_. This is the case for the current APU22 solution, discussed in the following subsection. For the χ_UND_ option, there is the additional challenge of knowing enough about the control of the undulator, which is not always the case, particularly with third-party instruments.

The last operation mode is the *triggered* mode, in which trajectories for *r*_BRG_ can be directly read from a file TRF_BRG_ that is pre-generated by the user. At bending-magnet beamlines, this may open possibilities in terms of fly-scans with non-standard trajectories. At undulator beamlines, in turn, it separates the undulator and the HD-DCM from the control perspective, preventing the issues of numerical representation, sample rates or noise that were just described in the *follower* mode. In this case, the undulator controller is also required to be able to switch between modes, according to an equivalent operation block OPN_UND_, and to store and run the setpoints from a file TRF_UND_, which must match those of the HD-DCM for energy tuning. Then, trigger and synchronization must be handled at the hardware level or via sufficiently fast/deterministic software implementation. Here, as well, sufficient reliability is needed in the undulator. This mode is unfortunately not yet available with the APU22, since an additional board is needed in the Beckhoff’s controller for external trigger capabilities. Yet, this feature is expected to be implemented and tested in the near future.

Finally, complementary performance estimators in Fig. 6 ▸ can be optionally computed in real-time to indicate compliance in the experiments. Firstly, the sensitive virtual source vertical shift χ_VSV_, as functions *f*_VSV_ of BRG, GAP and PTC, *i.e.* θ_B_, *g* and Δ*Rx*, can be calculated using (5) and (9), or their calibrated equivalent, and evaluated against the source size. Then, the mismatch χ_ENG_ in the energy tuning between the source and the HD-DCM, as functions *f*_ENG_ of BRG, UND and HAR, *i.e.* θ_B_, *p*_u_ and *n*_u_, can be computed using (1) and (14), or their calibrated equivalent, and compared with Γ_*E*_ [see Fig. 5 ▸(*c*)]. This should become common practice in the future to increase the maturity and the robustness of the experimental procedures.

### 4.3. Upgraded APU22 HD-DCM integration topology

Due to the inherent fly-scan capabilities of the HD-DCM, the known significant benefits related to fly-scan spectroscopy (elaborated in Section 5.3), and the inconvenient limitations found in the originally available *follower* operation that were mentioned in the previous subsection, integration alternatives started to be investigated. Then, while the absence of the Beckhoff’s triggering board prevented fly-scan spectroscopy operation in the *triggered* mode, a solution complementing the *follower* mode operation has been developed in-house. Now, differently from the previous condition, the new architecture allows the HD-DCM to work close to ideal scanning conditions, as shown in the next section.

**Figure 7:**
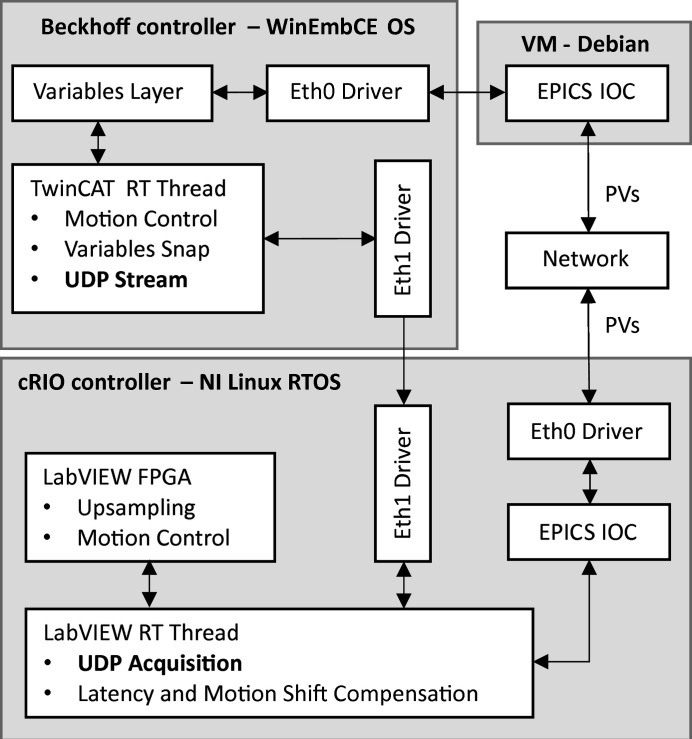
Upgraded integration topology between the APU22 and the HD-DCM at EMA and MANACÁ. The APU22 uses a Beckhoff controller with a Windows Embedded CE operating systems (OS) and an external virtual machine (VM) with a Debian OS for the EPICS IOC server. The HD-DCM is based on an NI linux real-time operating system (RTOS), with an embedded EPICS IOC server for EPICS process variables (PVs). A direct Ethernet (Eth) link using UDP (user datagram protocol) for setpoint streaming is now available for *follower* mode operation. Further details can be found in the text.

The upgraded topology is depicted in Fig. 7 ▸. As before, for standard communication with the external world, the Beckhoff’s controller, running a Windows Embedded CE operating system (WinEmbCE OS), exchanges variables via a Python layer (Variables Layer) and an ethernet port (Eth0 driver) with an external virtual machine (VM-Debian), which runs an EPICS IOC server for exchanging the EPICS PVs with the network. The cRIO controller, running a linux real-time operating system (NI Linux RTOS), in turn, has an embedded EPICS IOC layer [Nheengatu (Alnajjar *et al.*, 2019 ▸)], directly communicating with the network via an ethernet port (Eth0 driver).

Yet, now, the Beckhoff’s and the cRIO controllers are also directly connected via a unidirectional ethernet access (Eth1 drivers), which can stream the APU22 position setpoint *r*_UND_ to the HD-DCM (see Fig. 6 ▸). In the same motion control TwinCAT RT thread in the Beckhoff, motion variables (*e.g.* position and velocity) are calculated, sampled and streamed via UDP (user datagram protocol) through the dedicated ethernet link. At the cRIO side, LabVIEW RT code (as part of SEL_UND_ in Fig. 6 ▸) is responsible for decoding and treating the data.

Indeed, in spite of the peer-to-peer ethernet link and both implementations being on real-time threads, there are inherent delays, jitters and latencies in the system, which can be in principle characterized and partly compensated for in the LabVIEW RT code. One of these was the mechanical delay in the undulator response, thus allowing the HD-DCM to be closer to the real position rather than simply following an unrealistic reference setpoint. Moreover, given that the undulator control loop runs at 1 kHz only and the HD-DCM at 20 kHz, a LabVIEW FPGA code was implemented (also as part of the abstract SEL_UND_ block in Fig. 6 ▸) to generate an upsampled position reference to the HD-DCM control algorithm.

In this configuration, the Beckhoff’s controller is the one responsible for defining a suitable higher-order trajectory profile for smooth performances. Hence, obtaining deeper access and suitably defining Beckhoff’s internal variables for acceleration and jerk has also been an upgrade action, as compared with the early operation phase. Yet, as typical in high-performance mechatronic systems [see Butler *et al.* (2020 ▸)], a lot more can be expected to be investigated and optimized in terms of trajectories in the future, once other performance/operation bottlenecks at the beamline are eliminated. The following section demonstrates the latest progress and discusses the current operational limitations via concrete experimental examples.

## 5. Experimental results

Before becoming available to the general beamline user, the HD-DCM goes through a primary commissioning procedure that has been iteratively developed at the MANACÁ and the EMA beamlines at Sirius, according to the particularities of this new instrument and its integration system. As elaborated by Geraldes *et al.* (2021*a*) ▸, this includes: (1) *energy calibration* for the angular metrology of the Bragg rotary stage with respect to reference absorption edges; (2) *DCM-undulator tuning* between the Bragg angle and the undulator phase for the multiple harmonics (see also Section 3.2); and (3) *fixed-exit calibration* concerning the HD-DCM crystal cage (gap, pitch and roll). Then, part of these routines may be executed by the beamline crew or users via automated Python scripts on a regular basis, as sanity checks or re-calibration for improved accuracy. The first commissioning results of the HD-DCMs with X-rays at MANACÁ and EMA were described by Geraldes *et al.* (2021*a* ▸, 2022*c*) ▸. In the following subsections, updated results for stand-still and scanning performances at EMA, as well as methods developed for data analysis, are disclosed.

### 5.1. EMA beamline setup

**Figure 8:**
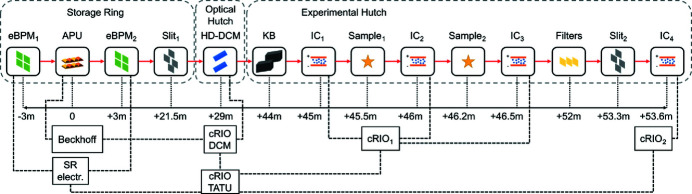
Simplified EMA beamline setup for the experiments with the HD-DCM, including: the APU22 source, electron beam position monitors (eBPMs); high-heat-load slits (Slit_1_); the HD-DCM; focusing Kirkpatrick–Baez (KB) mirrors; ionization chambers (IC_1_, IC_2_, IC_3_ and IC_4_); sample stages (Sample_1_ and Sample_2_); metal filters and monochromatic slits (Slit_2_). Connections among the Beckhoff’s motion controller for the APU22, storage ring (SR) electronics for the eBPMs, and NI’s cRIOs used as controller (HD-DCM), data acquisition hardware (ICs) and trigger unit (TATU) are also indicated. More details are provided in the text. Distances with respect to the undulator are provided for general reference.

The experimental setup layout is depicted in Fig. 8 ▸. The APU22 source, with its Beckhoff’s motion controller, is at the origin; electron beam position monitors (eBPMs) positioned 3 m upstream and downstream can provide transversal (*x* and *y*) and steering (pitch and yaw) data with sampling rates up to 572 kHz via dedicated electronics in the storage ring (SR); a set of high-heat-load slits (Slit_1_) is used to define the acceptance to the HD-DCM at 29 m; a set of bendable Kirkpatrick–Baez (KB) mirrors at 44 m is used to collimate or focus the downstream X-ray beam; two sample stages are present at 45.5 and 46.2 m; a set of pneumatic metal filters can be used at 52 m; a set of monochromatic slits is positioned at 53.3 m; finally, ionization chambers (IC_1_, IC_2_, IC_3_ and IC_4_) are used both before and after the samples for absorption experiments, as well as after the monochromatic slits (Slit_2_) for stability measurements.

The ICs were connected to Stanford Research Instruments’ SR570 low-noise current preamplifiers, whose voltages were then digitized at cRIO units (cRIO_1_ and cRIO_2_) using NI’s 16-bit NI-9215 analog input boards with 100 kHz sampling rate. Indeed, the cRIO has been chosen as one of the standard data acquisition hardware/controllers for Sirius beamlines, thus not only hosting the entire control of the HD-DCM but also being used to handle digital and analog signals of a variety of devices at rates up to 10 MHz. At the higher-level user operation, the so-called Nheengatu solution has been developed by the Beamline Software Group group (Alnajjar *et al.*, 2019) to integrate cRIO variables in LabVIEW into the EPICS framework. Moreover, a special software module, known as the time and trigger unit (TATU) (Piton *et al.*, 2021 ▸), has been developed in-house by the Beamline Software Group for the NI-9401 board to work as a synchronization unit in the microsecond range. This way, data from the eBPMs, the HD-DCM and the ICs can be already synchronized in the sub-millisecond domain using TTL (transistor–transitor logic) trigger signals. The APU22 is expected to become part of the complete triggering framework as soon as the complementary Beckhoff’s triggering board is commissioned.

### 5.2. Stand-still performance

Stand-still performances of the HD-DCM with pitch levels around 10 nrad RMS up to 2.5 kHz according to its embedded metrology have been reported by Geraldes *et al.* (2022*a* ▸) and Geraldes *et al.* (2022*c*) ▸ for the units at MANACÁ and EMA, respectively. With X-rays, however, only a preliminary stability evaluation attempt at MANACÁ has been reported so far by Geraldes *et al.* (2021*a*) ▸. Here, further analyses of the angular vibrations are carried out at EMA using both the rising edge of rocking curves and knife-edge methods, as described by Yamazaki *et al.* (2013 ▸), Chumakov *et al.* (2014 ▸) and Sergueev *et al.* (2016 ▸).

#### 5.2.1. Rocking curves

Starting with the rocking curves, the experiments were performed at 25.5 keV, *i.e.* close to the edge of Ag. This has been about the highest energy used so far at the beamline mainly due to the weak emission of the APU22 [see Fig. 4 ▸(*a*)] at higher energies, with the 13th harmonic being required for 25.5 keV. The selection of the highest possible energy was an attempt to maximize the sensitivity provided by narrower rocking curves [see Sergueev *et al.* (2016 ▸)]. The high-heat-load slits were set to 430 µm × 600 µm (v × h), providing an acceptance close to 15 µrad × 20 µrad, which offers a reasonable compromise between spectral purity for different diffraction orders and flux (see Fig. 4 ▸). Both Si(333) and Si(555) reflections were used, hence with Bragg angles around 13.45°and 22.81°. The measurements were taken with the IC_4_, having the monochromatic slits completely open, and the sample stages and the IC_2_ and IC_3_ removed from the beam. The IC_1_ was left in place to work as a filter for the lower-order reflections, being sufficient for the Si(333) measurements. For the Si(555) measurements, in turn, Al filters from the filter box were added to reduce the tails in the rocking curve, as shown next. The IC_1_ was filled with Ar95%/Kr5% and IC_4_ with N_2_40%/Ar60% for efficiency at 25.5 keV. Finally, the amplifier gains for the IC_4_ were set to 1 nA V^−1^ and 100 pA V^−1^ for the Si(333) and the Si(555) measurements, respectively.

**Figure 9:**
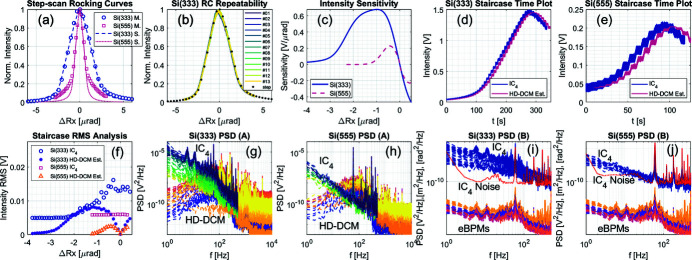
Rocking-curve-based stability assessment with the HD-DCM: (*a*) Si(333) and Si(555) rocking-curve measurements (M) via step-scans compared with simulations (S); (*b*) fly-scan rocking-curve repeatability test, with 13 measurements over about 2.5 h; (*c*) intensity sensitivity curves based on the derivative of the Si(333) and Si(555) rocking curves; (*d* and *e*) intensity staircase time plots of ionization chamber IC_4_ for Si(333) and Si(555), together with intensity variation estimates from the HD-DCM internal metrology (with a time offset of 10 s for readability); (*f*) root-mean-square (RMS) values from the steps of the staircase measurements and estimates; (*g* and *h*) power spectral density (PSD) from the steps of the staircase measurements and estimates; (*i* and *j*) PSD from staircase measurements together with electron beam position monitor (eBPM) data and IC_4_ noise floors [note: eBPM data in (*i*) borrowed from (*j*) measurements due to data loss, but without any loss of applicability]. For further details see text.

The step-scan rocking curve measurements, with integration time of 0.1 s per point and total measurement times around 1 min, are shown in Fig. 9 ▸(*a*). In spite of the residual tails from lower-order reflections, particularly in the Si(555) case, the agreement with the nominal simulated values (Sanchez del Rio *et al.*, 2011 ▸) is remarkable, with FWHMs of about 3 µrad and 1.12 µrad. Furthermore, even though the total power in the HD-DCM in these conditions is only of the order of 1 W, *i.e.* two orders of magnitude lower than the expected final load when the storage ring is at full current and the final undulator is installed, these results prove the high quality of the crystals [manufactured at the Advanced Photon Source (APS) by Elina Kazman’s team] and of the clamping and cryogenic cooling solutions [see Geraldes *et al.* (2022*a* ▸)]. Next to that, as a side evaluation of fly-scan options in pitch, repeatability and long-term stability, Fig. 9 ▸(*b*) shows a series of thirteen 10 s-long fly-scan measurements for the Si(333), taken every 12 minutes in a total of about 2.5 h. The results find good agreement with the initial step-scan, prove the internal metrology repeatability, and demonstrate stability over a few hours, apparently with only a small drift of about 0.1 µrad h^−1^.

Then, with the rocking curves, conversions between intensity and inter-crystal parallelism can be made for stability analyses. Fig. 9 ▸(*c*) shows the intensity sensitivity obtained for both step-scan measurements by taking the derivative of fitted peak functions (not shown) of the rocking curves. It is worth noting that, perhaps counter-intuitively, although the Si(555) has a steeper rising edge, the larger flux available with Si(333) made its sensitivity larger, even with a tenfold smaller amplifier gain. Besides that, the storage ring is not yet operating in top-up mode and its current during these measurements varied from 92 to 58 mA, respectively, thus reducing the potential sensitivity with Si(555) by 42% (with respect to the 100 mA injection level).

The actual stability experiments consisted of running staircase-like rocking curves, as proposed by Yamazaki *et al.* (2013 ▸). Hence, while making continuous acquisition at sampling rates of 1 kHz for the eBPMs and the IC_4_, and 20 kHz for the HD-DCM, the second crystal of the HD-DCM would start from a detuned condition and move over the rocking curves in a given number of steps. At every step, a dwell time of 5 s was taken for further statistical analyses. Figs. 9 ▸(*d*) and 9 ▸(*e*) show the time data for these scans. Together with the IC_4_ signal, estimated intensity levels for the HD-DCM are calculated using the high-quality internal metrology — which was consistently around 11 nrad RMS at all times — and the intensity sensitivity curves.

Next, the RMS values obtained in each step are summarized in Fig. 9 ▸(*f*). While the HD-DCM estimates replicate the sensitivity shapes, the IC_4_ signals show different results. For the Si(333) measurement, it leaves a base noise floor and starts to build up close to what might be interpreted as the HD-DCM contribution, but, instead of decreasing with the HD-DCM after the sensitivity peak, it continues to rise, in a closer approximation with the absolute intensity shape. For the Si(555) measurement, in turn, the RMS value simply remains at a constant noise floor, regardless of the absolute intensity value.

To further investigate these results, Figs. 9 ▸(*g*) and 9 ▸(*h*) provide the power spectral density (PSD) for each step in color gradients: the IC_4_, in the front and between 1 and 500 Hz, goes from light to dark (‘viridis’ colormap) as the pitch angle increases, whereas the HD-DCM, at the back and between 1 Hz and 10 kHz, goes from dark to light (‘plasma’ colormap). In neither case would it be expected that the HD-DCM estimate would overtake the IC_4_ spectra because, by definition, the HD-DCM is only one of the contributions that might affect the intensity measurements. Moreover, the most prominent peaks in the HD-DCM estimate do not appear as the most relevant ones in the IC_4_ spectra.

The fact is that even though the acquisition rate for the IC_4_ was 1 kHz, the large gains (≤1 nA V^−1^) required in these measurements were associated with low bandwidths of only 15 Hz for the Si(333) and 10 Hz for the Si(555) with the SR570 amplifier (as specified by the supplier). As a matter of fact, bandwidth limitations are often the case for most current amplifiers at large gains, as a way to handle spurious noise. Yet, with the Si(333) an increase in the IC_4_ spectra can be seen, with the intensity being dominated by the low-frequency contribution and by peaks at 60, 180 and 300 Hz. The Si(555), in turn, barely changes with the absolute intensity, being again dominated by peaks at 60, 180 and 300 Hz. This suggests that the first is sensitive to contributions from the source, whereas the latter is already limited by the noise in the detecting elements.

This can be further confirmed by including the noise levels (floors) that had been previously measured for the ICs in the setup without the beam, and by qualitatively comparing the IC_4_ spectra with those of eBPM signals, as shown in Figs. 9 ▸(*i*) and 9 ▸(*j*). Here, the multiple steps are represented by the same color that is associated with the different elements, namely: IC_4_ signal, electron beam horizontal position, electron beam vertical, electron beam yaw, electron beam pitch, and horizontal position of a dispersive eBPM (qualitatively related to the oscillation of the energy of the electrons in the storage ring, not shown in Fig. 8 ▸). In Fig. 9 ▸(*i*), the IC_4_ PSD is mostly above the noise floor, whereas the low-frequency behavior, the peaks at 6 and 12 Hz, and the ‘bump’ between 30 and 100 Hz do correlate with features in the eBPM signals, such that it becomes clear that the beam instabilities in the storage ring, with RMS values up to 2 µm and 1.5 µrad, are still truly affecting the intensity stability at the beamline. In Fig. 9 ▸(*j*), only the very lowest range of the Si(555) signal is above the noise floor, endorsing the discussion above.

Consequently, being close to 10 nrad RMS according to its internal metrology, the pitch stability of the HD-DCM seems indeed too small to be measurable via X-ray intensity over rocking-curve edges under the current conditions of the EMA beamline and Sirius storage ring. It should be emphasized, however, that the stability levels investigated with the HD-DCM are about 20 times lower than those measured by Sergueev *et al.* (2016 ▸) and more than 50 times lower than measured by Yamazaki *et al.* (2013 ▸). Still, the data analysis methodology proposed here allows for a systematic evaluation of the problem, demonstrating that the current practical measurement limitations span from instabilities in the source itself to the detecting elements, due not only to low signals (related to low fluxes with the APU22) but also to spurious electronic noise. Then, it can be used as a validation tool in following the required improvements in experimental conditions over time.

#### 5.2.2. Knife edge

**Figure 10:**
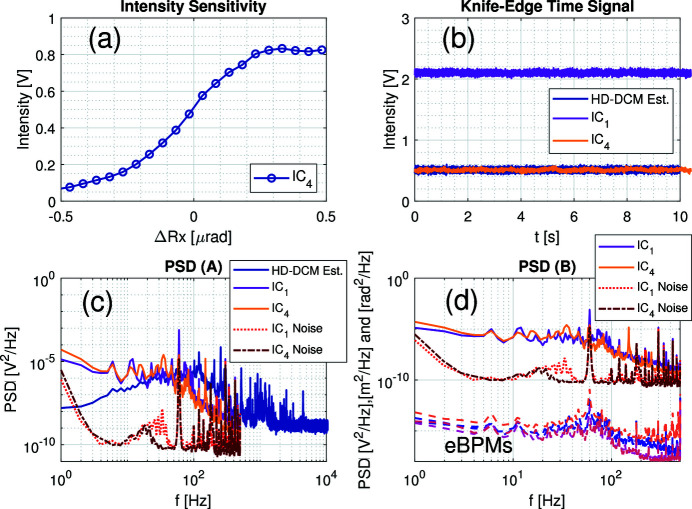
Knife-edge-based stability assessment with the HD-DCM: (*a*) second crystal pitch step-scan measurement for angle-to-intensity mapping using ionization chamber IC_4_; (*b*) intensity measurement at maximum sensitivity point, with reference ionization chamber IC_1_, transmitted signal IC_4_ and intensity variation estimate from the HD-DCM internal metrology; (*c*) power spectrum density (PSD) from the knife-edge measurement, including the HD-DCM estimate and IC noise floors; and (*d*) PSD from knife-edge measurements with electron beam position monitor (eBPM) data and IC noise floors. For further details see text.

Next, taking advantage of the experimental setup with the Si(333) configuration, an equivalent stability analysis was carried out with a knife-edge method. Firstly, the beam was focused with the KB set to about 7 µm × 10 µm (v × h) at the monochromatic slits. Then, with the crystals tuned at the peak of the rocking curve, the bottom blade of the slit was scanned in front of the beam and finally centered halfway for maximum sensitivity. Next, the second crystal of the HD-DCM was step-scanned in pitch to obtain the intensity sensitivity curve shown in Fig. 10 ▸(*a*). Lastly, the second crystal was repositioned at the tuned condition (maximum sensitivity) and continuous data were collected for 10 s.

Fig. 10 ▸(*b*) shows the time data, with the IC_4_ signal, the estimated intensity level for the HD-DCM (derived from its internal metrology signal and the sensitivity curve) and also the IC_1_, which was used as a reference signal with a gain of 5 nA V^−1^. At the top of the rocking curve, the HD-DCM should have nearly zero impact in the IC_1_, which is before the knife and also takes the Si(111) background flux. Besides that, equivalently to the staircase measurements, under ideal experimental conditions the distribution of the IC_4_ could not possibly be smaller than that of the HD-DCM.

Turning once again to the frequency domain, Figs. 10 ▸(*c*) and 10 ▸(*d*) show the PSDs comparing all signals and including the noise floors from dark conditions. In Fig. 10 ▸(*c*), it can be seen that the ICs are well above the noise level for most of the frequencies. Yet, they surprisingly show very similar levels and behavior between them, from which any contributions from the HD-DCM can be hardly recognized. Only the largest peaks from the HD-DCM at 120, 204 and 255 Hz seem to find counterparts in the IC_4_, but with 1000-fold reduced amplitudes. Then, from Fig. 10 ▸(*d*), both ICs take the structures from the eBPMs spectra, as also discussed for Fig. 9 ▸(*i*), which again limited the investigation of the inter-crystal pitch stability of the HD-DCM with X-rays. Still, the sensitivity peak here reached 2 V µrad^−1^ (not shown), which is 2.5 times larger than what was achieved with the Si(333) rocking edge [see Fig. 9 ▸(*c*)], making this the most sensitive and promising method available at EMA at the moment.

Unfortunately, the beam time was over before a knife-edge measurement with Si(111) could be performed. Indeed, since this method does not rely on the sharpness of the rocking curve, working with the first diffraction order would allow fluxes one or two orders of magnitude larger (with a broader Darwin width), which could use lower gains (≫1 nA V^−1^) and reach bandwidths of hundreds of Hz to a few kHz, while improving the signal-to-noise ratio. In that sense, lower energies at lower harmonics should also be explored, as more than two extra orders of magnitude might be gained in flux [see Fig. 4 ▸(*a*)]. Nevertheless, as discussed by Sergueev *et al.* (2016 ▸), one of the drawbacks of the knife-edge concept is its sensitivity to the mechanical stability of other elements in the beamline, including the mirrors and the knife itself. For example, in Figs. 10 ▸(*c*) and 10 ▸(*d*) the sharp peak at 150 Hz in IC_4_ is likely to be related to an unmapped mechanical resonance in the optical chain. These contributions may become more relevant once the other dominant effects are eliminated.

To conclude, although the replacement date of the official undulator source — which will improve the conditions for pitch stability measurements based on rocking curve edges — remains unclear, significant improvements in overall stability are expected already in the short term. These include: (1) the implementation of the FOFB system, which will extend the electron beam control bandwidth well beyond the 1 Hz that is currently provided by the slow orbit feedback (SOFB) system (Marques *et al.*, 2022 ▸); (2) the top-up operation mode at the storage ring, which should minimize current-related transients; and (3) a refined grounding work to reduce electronic noise at EMA. Other than that, this commissioning round fostered the development of this systematic data analysis pipeline and a refinement in the real-time integration between several components of the storage ring and the beamline, which proved to be decisive in interpreting the experimental data and will be essential for fast experiments with sufficient statistics. A new round of beam time for stand-still stability assessment of the HD-DCM will be realized after the identified dominant sources of beam variation and noise are solved.

### 5.3. Scanning performances

In spite of the electron beam instabilities and electronic noise levels that limited the evaluation of the HD-DCM performance in the stand-still experiments with X-rays, EMA has been perfectly able to host users from the scientific community and carry out relevant spectroscopy experiments on a regular basis. The following subsections provide results showing the remarkable motion performance and spectroscopy capabilities currently available at EMA.

#### 5.3.1. Fly-scan motion evaluation

**Figure 11:**
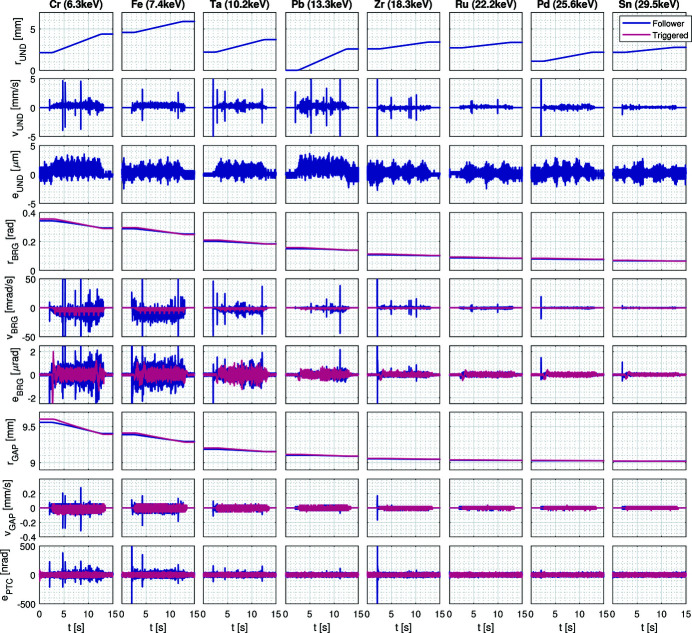
Performance varibles in 1 keV fly-scans taking about 15 s with the HD-DCM with Si(111). A selection of energies covering the current useful range at the EMA beamline and multiple harmonics in the APU22 is made. Together with the central energy of the scan, elements of interest with absorption edges within these ranges are indicated. Variables of the undulator (UND), the Bragg angle control loop (BRG), and the crystal cage loops gap (GAP) and pitch (PTC) are shown for a comprehensive motion performance evaluation. The upgraded *follower* performance can be directly compared with independend *triggered* mode as a proof of concept. The complete discussion is provided in the text.

The current possibilities in energy fly-scans, with the HD-DCM in the *follower* mode and the upgraded integration topology with the APU22 (see Section 4.3), are exemplified in Fig. 11 ▸. Performance variables in 1 keV scans taking about 15 s with Si(111) are shown for a selection of energies for elements of interest covering the currently useful range of energies at EMA. The undulator reference signal *r*_UND_ entering the HD-DCM control loops is shown to cover 1 to 2 mm over different harmonics for the different energies. The undulator velocity *v*_UND_, obtained by differentiating *r*_UND_, is bound generally below 1 mm s^−1^, except for larger random peaks found in most of the scans. Their cause seems to be related to discontinuities in the trajectory generated by the Beckhoff’s controller, but this issue is still under investigation for a definitive solution. Yet, although they partly degrade the HD-DCM loops, as discussed below, this is to a far less extent than the degradation observed with the previous architecture [see Geraldes *et al.* (2021*b* ▸, 2022*c* ▸)]. Regarding the undulator tracking error *e*_UND_, it shows good performance, with deviations of ≤3 µm, thus obeying the phase error tolerances 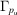 specified in Fig. 5 ▸(*d*).

The amplitude of the Bragg angle reference signal *r*_BRG_ decreases with energy, going from a stroke of 0.05 rad (2.85°) to 0.002 rad (0.1°) only. The angular velocity *v*_BRG_ is generally bound to ≤30 mrad s^−1^ (1.7° s^−1^), but the reaction to the peaks in the undulator velocity is clear. The control error *e*_BRG_ is also significantly reduced as the photon energy increases, typically going from about 1 µrad (in the cruising range outside the acceleration/deceleration regions) to less than 0.2 µrad at high energies. Naturally, the influence of the undulator velocity bursts is again obvious. These angular errors can be converted to energy variations according to (3), here falling <0.1 eV. Their particular impact on the experiment depend on the contributions within the integration times. More details about this will be provided in another publication under preparation.

Regarding crystal cage loops, the amplitude of the gap reference signal *r*_GAP_ also decreases with energy, going from a stroke of 0.15 mm to 0.001 mm only. The gap velocity *v*_GAP_ is generally bound to ≤0.1 mm s^−1^, mostly to dynamically correct the gap error (∣*e*_GAP_∣ ≤ 10 nm, not shown), since the gap stroke for these energies are low, except during the reactions to undulator peaks. Finally, the control error for the pitch between crystal *e*_PTC_ is always within 15 nrad RMS, even at the lower energies and with the disturbances of the undulator signal. In these experiments, the destination position, velocity and motion trigger were sent to the Beckhoff’s controller via PVs, and the trajectories had constant speed in the undulator coordinates. For trajectories linear in energy, pre-calculated vectors must be passed to the APU22 controller, a functionality not yet validated, but that is expected to become available soon.

Still, to remove any doubts about the undulator signal being responsible for the residual performance degradation in Fig. 11 ▸, it also shows the Bragg and crystal cage variables for similar trajectories with the HD-DCM in the *triggered* mode, *i.e.* following a pre-planned trajectory and completely decoupled from the undulator. Small differences in the reference signals are because the *follower* mode used polynomials (*f*_BRG_ in Fig. 6 ▸) calibrated at the beamline, whereas the *triggered* mode tests used nominal polynomials for this undulator. It can be seen that *e*_BRG_ and *e*_PTC_ are close to ideal performance, at ≤0.35 µrad and ≤15 nrad RMS (at 20 kHz), respectively.

The undulator could not make part of this last proof of concept because, as mentioned above, a functional board is still missing in the Beckhoff’s controller to allow for external triggers (and integration with the TATU framework). As soon as it is available, it will be possible to compare the results from the current streaming implementation with fully triggered performance for a definitive beamline operation choice. It should be noted that this high-performance synchronization effort is in agreement with what has been developed in other synchrotrons (Hidas *et al.*, 2022 ▸), but here with additional subtleties related to the large sensitivity of the HD-DCM as a high-performance mechatronic system.

To conclude, the choice of scans within 15 s was arbitrary and tests at least twice as fast have achieved comparable results (not shown). Naturally, at some point, higher accelerations and faster motions on bearings, together with larger strokes required for lower energies, start to increase disturbances. These will require case-by-case analyses, particularly as the lowest energy range of the HD-DCM starts to be explored after the replacement of the APU22.

#### 5.3.2. Spectroscopy results

The X-ray absorption spectroscopy (XAS) technique is a powerful tool for studying the electronic behaviors of materials under extreme thermodynamical conditions, such as high pressure and low temperatures. In particular, high-pressure studies are especially interesting for the 5*d* transition metals, whose energy levels involved at the *L*_2,3_ edges are above 10 keV, therefore compatible with diamond anvil cells (DACs) (Itié *et al.*, 2016 ▸). This kind of study is among the central scientific goals of the EMA beamline at Sirius.

**Figure 12:**
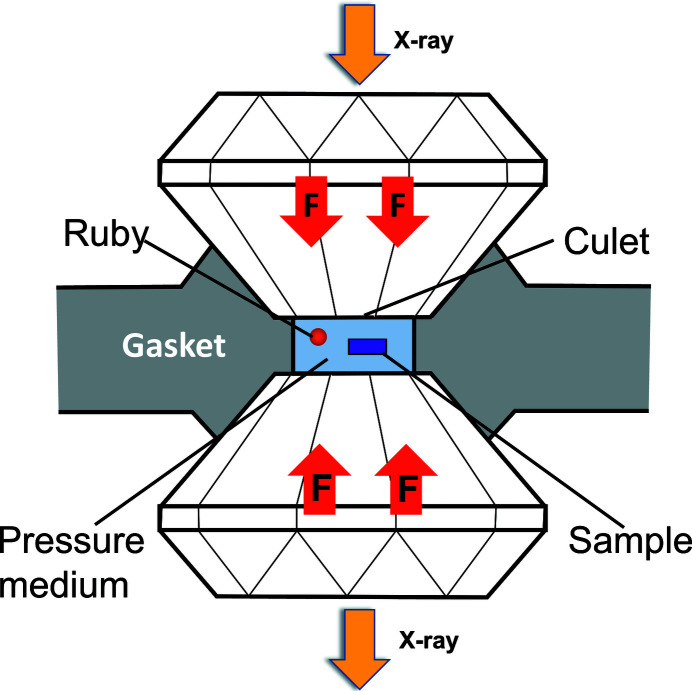
High-pressure setup using diamond anvil cells (DACs) to submit samples to extreme conditions. The focused X-ray beam goes through the two diamond cells that are loaded against each other through a gasket to create a closed environment with pressures reaching several tens to hundreds of GPa. A pressure medium is used to distribute the pressure and a ruby sphere is used as a pressure gauge via an optical signal. Culet sizes are typically of the order of a few hundred micrometres.

Here, to evaluate the spectroscopy capabilities with the HD-DCM during the EMA scientific commissioning, a high-pressure XAS experiment was carried out with the Si(111) set of crystals at the Pt *L*_2_ and *L*_3_ edges (2*p* → 5*d* transition) to probe the pressure evolution of valence band spin–orbit coupling of Pt metal. The measurements, at room temperature, were performed in transmission geometry, as illustrated in Fig. 12 ▸, using a membrane-driven copper–beryllium DAC prepared with two full anvils, with a culet size of 350 µm to reach pressures as high as 60 GPa. A 5 µm-thick platinum foil was loaded in a 60 µm hole of a rhenium gasket, together with a 5 µm ruby sphere for *in situ* pressure calibration, and neon gas was used as a pressure-transmitting medium. Referring to Fig. 8 ▸, the DAC was mounted at the Sample_1_ position, where the beam was focused to 1 µm × 2 µm (v × h), whereas a Pt standard foil (Exafs Materials) was placed at Sample_2_, while IC_1_, IC_2_ and IC_3_ were used for intensity measurements.

The experiment consisted of incrementally increasing the sample pressure in steps of roughly 5 GPa, and, in each pressure step, measuring three spectra in step-scan mode followed by ten spectra in fly-scan mode, for both the *L*_2_ and *L*_3_ edges. The total energy range was 490 eV in all cases. For the step mode, energy step sizes of 0.5 eV were used around the edge (30 eV before and 30 eV after it) and 7 eV for the pre- and post-edge regions. The step-scan integration time was 0.5 s, and the total time for each spectra was about 6 min, with most of it consumed by overheads from start/stop actions, as also mentioned by Hidas *et al.* (2022 ▸). The fly-scans, in turn, were performed with 0.1 mm s^−1^ phase speed in the APU22 and 20 kHz sampling frequency for the HD-DCM and the ICs cRIOs (see Fig. 8 ▸), with a measurement time of 27 s per fly-scan spectrum.

**Figure 13:**
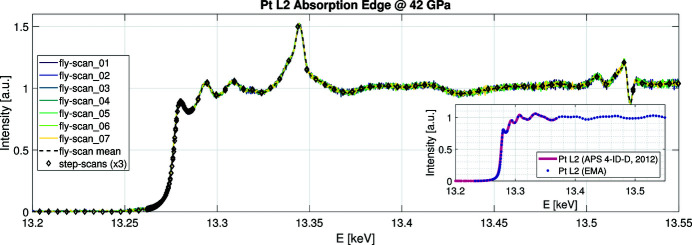
XAS spectra of a Pt foil under 42 GPa in a diamond anvil cell setup at EMA. Measurements over the Pt *L*_2_-edge were repeated ten times in fly-scan (27 s each) (three runs lost due to a vacuum interlock fault at the beamline) and three times in step-scan (6 min each), demonstrating the high-performance spectroscopy capabilities available at EMA with the HD-DCM and the APU22 source. The inset shows a simultaneous reference measurement of standard Pt sample, being compared with a previous measurement at the APS 4-ID-D beamline. Further discussion is provided in the text.

One representative set of data, for the *L*_2_ edge at 42 GPa, is depicted in Fig. 13 ▸. The colorful lines in the back (‘viridis’ colormap) are the fly-scan measurements, for which the effective sampling rate was averaged down to 40 Hz to improve the signal-to-noise ratio while achieving an energy resolution of 0.5 eV; the black dashed line is their average; the diamond markers show the points of three step-scan measurements, which overlap in most cases. The accurate correspondence between the two measurement methods demonstrates the reliability of the HD-DCM, the effectiveness of the upgraded *follower* mode operation, and the potential of the fly-scan method, with maximum practical speeds currently limited mostly by the APU22 flux and spurious electronic noise in the detection electronics (which is currently under improvement). Furthermore, it was found that the dispersion in the intensity signal of the fly-scan measurements is, in this case, dominated by a 12 Hz contribution (not shown in detail for conciseness), which was also observed in the stability measurements in Figs. 9 ▸ and 10 ▸, being likely caused by the instabilities in the electron beam. Again, these should be greatly improved with the implementation of the FOFB system.

It should be noted that the high-pressure XAS experiments using diamond anvil cells are particularly challenging because of the presence of diamond Bragg peaks in the spectra, such as those around 13.34 keV and above 13.5 keV. Although these Bragg peaks, inherent to the high-pressure XAS experiments, do bring special complications to the data analysis, for our purposes they reinforce the accuracy of the fly-scan as being identical to the step-scan along the entire energy range. Moreover, in addition to the drastic time efficiency increase, it becomes clear that the fly-scans have benefits related to continuous measurements, such as the wiggle feature close to 13.52 keV, which might be misinterpreted in a step-scan with a poorer resolution. As a further validation check, the inset in Fig. 13 ▸ compares a step-scan spectrum of the standard Pt foil in Sample_2_ (collected simultaneously with one of the step-scan measurements in the main plot) with a standard Pt *L*_2_ XAS spectrum that had been measured at the APS 4-ID-D beamline in 2012, where the same features can be identified.

**Figure 14:**
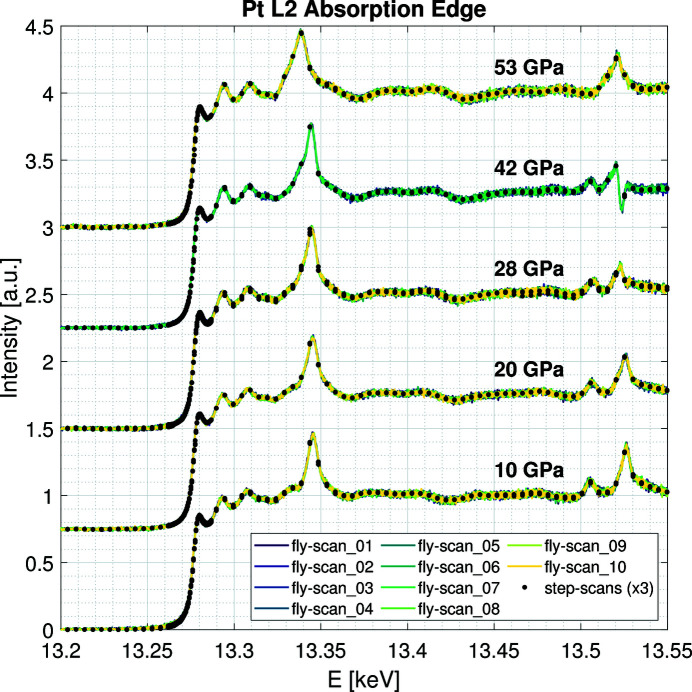
XAS spectra of a Pt foil under multiple high-pressure conditions in a diamond anvil cell setup at EMA, validating a high degree of reliability and repeatability of the HD-DCM and the beamline over hundreds of step- and fly-scan repetitions in a total experimental time around 36 h. Vertical offsets have been introduced for readability. Further discussion is provided in the text.

Finally, evaluating the robustness and repeatability of the experimental system, Fig. 14 ▸ shows five sets of measurements for the *L*_2_ edge from 10 to 53 GPa, with the step-scans now represented by solid black dots and vertical offset between the sets for readability. The changes in the signature of the spectra as a function of pressure can be promptly identified, but the same aspects emphasized for Fig. 13 ▸ hold true. The whole measurement time was approximately 36 h, including injection windows, since the storage ring is still not operating in top-up mode. Without any further compensations, the variation of the peak of the *L*_2_ absorption edge derivatives for all spectra (not shown), *i.e.* for more than 300 scans and including the current noise and electron beam stability limitations, was within ±0.75 eV. Also, considering that for each pressure step the HD-DCM would travel to the *L*_3_ edge (around 11.5 keV) and back, this proves the high degree of reliability achieved at EMA.

## 6. Conclusions

The efforts in designing and implementing the High-Dynamic Double-Crystal Monochromator from scratch, as a high-end mechatronic machine for Sirius new-generation beamlines, have been honored with unmatched fixed-exit and fly-scan spectroscopy capabilities. Indeed, the well known inter-crystal pitch stability performance bottleneck in standard DCMs is in the HD-DCM solved thanks to an improvement level varying from about 5- to more than 100-fold, since 10 nrad RMS (1 Hz to 2.5 kHz) can be achieved even during fast fly-scans, which, in addition, can also reduce measurement times by at least one or two orders of magnitude.

This article has summarized, from a new perspective, engineering and operational aspects that acquire critical relevance in enabling next-level experimental possibilities with the HD-DCM. The multi-axis and highly non-linear motion control problem is stated and an energy-tuning evaluation method, based on wave-propagation simulations, is also proposed to define positioning tolerances between the HD-DCM and the APU source. Then, limitations in the originally available integration architecture with the APU — which could have been actually predicted and prevented if a dedicated analysis had been carried out during the procurement phase of the commissioning undulators — have been overcome with a new control topology, that has been already able to provide close-to-ideal motion performance results. Furthermore, it was demonstrated how sensitive fast-acquisition experiments are to the whole beamline system, from the stability of the electron beam in the storage ring to high-bandwidth noise levels on detectors. Hence, the essential role of a holistic approach in integrating the HD-DCM to the beamlines, so as to achieve ultimate performances, higher efficiency and maximum throughput in fourth-generation light-source beamlines, cannot be emphasized enough. Still, scientific commissioning results demonstrating operational reliability and already tenfold faster fly-scan XAS measurements are discussed.

Technical commissioning of the HD-DCM will proceed in parallel with beamline operation, as the parameters and functional systems of the storage ring gradually reach final values, and the definitive undulators are installed at the beamlines. These will provide higher electron beam stability and higher fluxes, eventually enabling practical experiments with faster acquisition rates than today, but also affecting the HD-DCM with higher power loads. Moreover, as a next step, spectroscopy performance in *triggered* mode will be compared with the current solution in *follower* mode also with X-rays. Finally, a new HD-DCM model for even faster scans, the so-called HD-DCM-Lite, is now under assembly phase for two new beamlines, and first results are expected in early 2023.

## Figures and Tables

**Table 1:** List of abbreviations used in this paper

APS	Advanced Photon Source	MANACÁ	Macromolecular Micro and Nano Crystallography
APU	Adjustable-phase undulator	MIMO	Multiple-input–multiple-output
BRG	Bragg angle control loop	OPN	Operation mode selector
CCG	Crystal cage	OS	Operating system
CNPEM	Brazilian Center for Research in Energy and Materials	PTC	Inter-crystal pitch control loop
cRIO	CompactRIO	PSD	Power spectral density
DAC	Diamond anvil cell	PV	EPICS process variable
DCM	Double-crystal monochromator	RLL	Inter-crystal roll control loop
eBPM	Electron beam position monitor	RMS	Root mean square
EMA	Extreme methods of analysis	RT	Real-time
EPICS	Experimental Physics and Industrial Control System	SEL	Operation selector
FWHM	Full width at half-maximum	SOFB	Slow orbit feedback
ESRF	European Synchrotron Radiation Facility	SISO	Single-input–single-output
FOFB	Fast orbit feedback	SR	Storage ring
FPGA	Field-programmable gate array	*SRW*	Synchrotron Radiation Workshop
GAP	Inter-crystal gap control loop	TATU	Trigger and time unit
HAR	Undulator harmonic variable	TRF	Trajectory file control block
HD-DCM	High-Dynamic DCM	TRG	Trajectory generator control block
IC	Ionization chamber	TTL	Transistor–transistor logic
IOC	EPICS Input/Output Controller	UDP	User datagram protocol
IVU	In-vacuum undulator	UND	Undulator control loop
KB	Kirkpatrick–Baez mirror configuration	VM	Virtual machine
LNLS	Brazilian Synchrotron Light Laboratory	XAS	X-ray absorption spectroscopy

## References

[bb1] Alnajjar, D. A. D., Fedel, G. S. & Piton, J. R. (2019). *Proceedings of the 17th International Conference on Accelerator and Large Experimental Control Systems (ICALEPCS2019)*, New York, NY, USA, pp. 997–1000. WEMPL002.

[bb2] Ashcroft, N. W. (2003). *Solid State Physics.* Thomson Press.

[bb3] Baker, R., Baboulin, D., Barrett, R., Bernard, P., Berruyer, G., Bonnefoy, J., Brendike, M., Brumund, P. M., Dabin, Y., Ducotté, L., Gonzalez, H., Malandrino, G., Marion, P., Mathon, O., Roth, T. & Tucoulou, R. (2018). *Proceedings of the 10th International Conference on Mechanical Engineering Design of Synchrotron Radiation Equipment and Instrumentation (MEDSI 2018)*, 25–29 June 2018, Paris, France, pp. 440–444. FROAMA07.

[bb25] Butler, H., Heertjes, M. F., Dirkx, N., van der Meulen, S., Ahlawat, R., O’Brien, K., Simonelli, J., Teng, K. & Zhao, Y. (2020). *2020 American Control Conference*, 1–3 July 2020, Denver, CO, USA, pp. 3686–3703. IEEE.

[bb4] Caliari, R. M., Geraldes, R. R., Moraes, M. A. L., Moreno, G. B. Z. L., Faassen, R., Ruijl, T. A. M. & Schneider, R. M. (2018). *Proceedings of the 16th International Conference on Accelerator and Large Experimental Control Systems (ICALEPCS’17)*, 12–16 October 2017, Barcelona, Spain, pp. 997–1002. TUSH203.

[bb5] Caliari, R. M., Geraldes, R. R., de Moraes, M. A. L. & Witvoet, G. (2020). *ASPE 2020 Spring – Design and Control of Precision Mechatronic Systems*, 6–8 May 2020, pp. 125–130. American Society for Precision Engineering.

[bb6] Chubar, O. & Elleaume, P. (1998). *Proceedings of the Sixth European Particle Accelerator Conference (EPAC98)*, 22–26 June 1998, Stockholm, Sweden, pp. 1177–1179.

[bb7] Chumakov, A. I., Sergeev, I., Celse, J.-P., Rüffer, R., Lesourd, M., Zhang, L. & Sánchez del Río, M. (2014). *J. Synchrotron Rad.* **21**, 315–324. 10.1107/S1600577513033158 24562552

[bb8] Cowan, P. L., Hastings, J. B., Jach, T. & Kirkland, J. P. (1983). *Nucl. Instrum. Methods Phys. Res.* **208**, 349–353.

[bb9] Dolbnya, I. P., Sawhney, K. J. S., Scott, S. M., Dent, A. J., Cibin, G., Preece, G. M., Pedersen, U. K., Kelly, J. & Murray, P. (2019). *J. Synchrotron Rad.* **26**, 253–262. 10.1107/S1600577518014662 PMC6337885 30655493

[bb11] Eriksson, M., van der Veen, J. F. & Quitmann, C. (2014). *J. Synchrotron Rad.* **21**, 837–842. 10.1107/S1600577514019286 25177975

[bb13] Fan, Y., Qin, H., Zhu, W., Jia, W., Liu, Y., Wang, J. & Li, Z. (2020). *Nucl. Instrum. Methods Phys. Res. A*, **983**, 164636.

[bb15] Geraldes, R. R., Caliari, R. M., Moreno, G. B. Z. L., Ronde, M. J. C., Ruijl, T. A. M. & Schneider, R. M. (2017*b*). *Proceedings of the 9th Mechanical Engineering Design of Synchrotron Radiation Equipment and Instrumentation (MEDSI 2016)*, 11–16 September 2016, Barcelona, Spain, pp. 44–47. MOPE19.

[bb14] Geraldes, R. R., Caliari, R. M., Moreno, G. B. Z. L., Sanfelici, L., Saveri Silva, M., Souza Neto, N. M., Tolentino, H. C. N., Westfahl Jr, H., Ruijl, T. A. M. & Schneider, R. M. (2017*a*). *Proceedings of the 9th Mechanical Engineering Design of Synchrotron Radiation Equipment and Instrumentation (MEDSI 2016)*, 11–16 September 2016, Barcelona, Spain, pp. 141–146. TUCA05.

[bb16] Geraldes, R. R., Caliari, R. M., Moreno, G. B. Z. L., Sanfelici, L., Saveri Silva, M., Tolentino, H. C. N., Westfahl Jr, H., Ruijl, T. A. M. & Schneider, R. M. (2018). *Proceedings of the 10th International Conference on Mechanical Engineering Design of Synchrotron Radiation Equipment and Instrumentation (MEDSI 2018)*, 25–29 June 2018, Paris, France, pp. 147–152. WEOAMA01.

[bb18] Geraldes, R. R., de Brito Neto, J. L., Caliari, R. M., Eleoterio, M. A. S., Luiz, S. A. L., Moraes, M. A. L., Perna, A. V., Silva, M. S. & de Albuquerque, G. S. (2021*a*). *Proceedings of the 11th International Conference on Mechanical Engineering Design of Synchrotron Radiation Equipment and Instrumentation (MEDSI 2020)*, 26–29 July 2021, Chicago, IL, USA, pp. 25–28. MOPB03.

[bb19] Geraldes, R. R., de Brito Neto, J. L., Coelho, E. P., Do Carmo, L. P., Horita, A. Y., Luiz, S. A. L. & Moraes, M. A. L. (2021*b*). *Proceedings of the 18th International Conference on Accelerators and Large Experimental Physics Control Systems (ICALEPCS2021)*, 14–22 October 2021, Shanghai, China, pp. 370–375. TUPV004.

[bb22] Geraldes, R. R., de Brito Neto, J. L., Saveri Silva, M., de Albuquerque, G. S., Luiz, S. A. L., Pinto, A. C., Rigamonti Jr, H. & Eleotério, M. A. S. (2022*c*). Presented at *14th International Conference on Synchrotron Radiation Instrumentation (SRI 2021)*, 28 March–1 April 2021, Hamburg, Germany.

[bb17] Geraldes, R. R., Moraes, M. A. L., Caliari, R. M. & Witvoet, G. (2020). *ASPE 2020 Spring – Design and Control of Precision Mechatronic Systems*, 6–8 May 2020, pp. 119–124. American Society for Precision Engineering.

[bb21] Geraldes, R. R., Moraes, M. A. L., Witvoet, G. & Vermeulen, J. P. M. B. (2022*b*). *Precis. Eng.* **77**, 90–103.

[bb20] Geraldes, R. R., Witvoet, G. & Vermeulen, J. P. M. B. (2022*a*). *Precis. Eng.* **77**, 110–126.

[bb23] Golovchenko, J. A., Levesque, R. A. & Cowan, P. L. (1981). *Rev. Sci. Instrum.* **52**, 509–516.

[bb24] Hastings, J. B. (1977). *J. Appl. Phys.* **48**, 1576–1584.

[bb26] Hettel, R. (2014). *J. Synchrotron Rad.* **21**, 843–855. 10.1107/S1600577514011515 25177976

[bb27] Hidas, D., Kiss, A. M., Rakitin, M., Sinsheimer, J., Tanabe, T. & Musardo, M. (2022). *Nucl. Instrum. Methods Phys. Res. A*, **1031**, 166505.

[bb28] Hussain, Z., Umbach, E., Shirley, D. A., Stöhr, J. & Feldhaus, J. (1982). *Nucl. Instrum. Methods Phys. Res.* **195**, 115–131.

[bb29] Itié, J. P., Baudelet, F. & Rueff, J.-P. (2016). *High Pressure XAS, XMCD and IXS*, ch. 14, edited by J. A. Van Bokhoven & C. Lamberti, pp. 385–705.

[bb30] Kelly, J., Lee, T., Alcock, S. & Patel, H. (2013). *J. Phys. Conf. Ser.* **425**, 052009.

[bb31] Koningsberger, D. & Prins, R. (1988). *X-ray Absorption: Principles, Applications, Techniques of EXAFS, SEXAFS and XANES.* Wiley-Interscience.

[bb32] Kristiansen, P., Horbach, J., Döhrmann, R. & Heuer, J. (2015). *J. Synchrotron Rad.* **22**, 879–885. 10.1107/S1600577515005664 PMC4489531 26134790

[bb33] Lemonnier, M., Collet, O., Depautex, C., Esteva, J. & Raoux, D. (1978). *Nucl. Instrum. Methods*, **152**, 109–111.

[bb34] Liu, L., Alves, M. B., Oliveira, A. C. S., Resende, X. R. & de Sá, F. H. (2021). *Proceedings of the 12th International Particle Accelerator Conference (IPAC2021)*, 24–28 May 2021, Campinas, Brazil, pp. 13–18. MOXA03.

[bb35] Marques, S. R., Alves, M. B., Arroyo, F. C., Calcanha, M. P., Canova, H. F., Limeira, B. E., Liu, L., Neuenschwander, R. T., Pereira, A. G. C., Tavares, D. O., de Sá F. H., Brunheira, G. O., Cardoso, A. C. T., Cardoso, R. B., Junqueira Leão, R., Leão, L. R., Martins, P. H. S., Moreira, S. S., Oliveira Neto, R. & Siqueira, M. G. (2022). *Proceedings of the 13th International Particle Accelerator Conference (IPAC2022)*, 12–17 June 2022, Bangkok, Thailand, pp. 226–229. MOPOPT002.

[bb36] Moraes, M. A. L., Caliari, R. M. & Geraldes, R. R. (2020). *ASPE 2020 Spring – Design and Control of Precision Mechatronic Systems*, 6–8 May 2020, pp. 131–136. American Society for Precision Engineering.

[bb37] Moreno, G. B. Z. L., Caliari, R. M., Geraldes, R. R. & Moraes, M. A. L. (2018). *Proceedings of the 16th International Conference on Accelerator and Large Experimental Control Systems (ICALEPCS2017)*, 12–16 October 2017, Barcelona, Spain, pp. 1941–1946. THPHA214.

[bb38] Onuki, H. & Elleaume, P. (2002). *Undulators, Wigglers and their Applications.* CRC Press.

[bb39] Piton, J. R., Alnajjar, D. A. D., Araujo, D. H. C., Brito Neto, J. L., do Carmo, L. P., Guedes, L. C. & Moraes, M. A. L. (2021). *Proceedings of the 18th International Conference on Accelerators and Large Experimental Physics Control Systems (ICALEPCS2021)*, 14–22 October 2021, Shanghai, China, pp. 908–911. THPV021.

[bb40] Sanchez del Rio, M., Canestrari, N., Jiang, F. & Cerrina, F. (2011). *J. Synchrotron Rad.* **18**, 708–716. 10.1107/S0909049511026306 PMC3267628 21862849

[bb41] Saveri Silva, M., Geraldes, R. R., Gilmour, A., Ruijl, T. A. M. & Schneider R. M. (2017). *Proceedings of the 9th Mechanical Engineering Design of Synchrotron Radiation Equipment and Instrumentation (MEDSI 2016)*, 11–16 September 2016, Barcelona, Spain, pp. 194–197. TUPE15.

[bb42] Sergueev, I., Döhrmann, R., Horbach, J. & Heuer, J. (2016). *J. Synchrotron Rad.* **23**, 1097–1103. 10.1107/S1600577516011188 27577762

[bb43] Sterbinsky, G. E. & Heald, S. M. (2021). *J. Synchrotron Rad.* **28**, 1737–1746. 10.1107/S1600577521008626 34738927

[bb44] Waterstradt, T., Diete, W., Schacht, A., Engblom, C., Scheel, M. & Weitkamp, T. (2018). Presented at *10th International Conference on Mechanical Engineering Design of Synchrotron Radiation Equipment and Instrumentation (MEDSI 2018)*, 25–29 June 2018, Paris, France. Poster WEPH42.

[bb45] Yamazaki, H., Ohashi, H., Senba, Y., Takeuchi, T., Shimizu, Y., Tanaka, M., Matsuzaki, Y., Kishimoto, H., Miura, T., Terada, Y., Suzuki, M., Tajiri, H., Goto, S., Yamamoto, M., Takata, M. & Ishikawa, T. (2013). *J. Phys. Conf. Ser.* **425**, 052001.

[bb46] Zhang, L., Sánchez del Río, M., Monaco, G., Detlefs, C., Roth, T., Chumakov, A. I. & Glatzel, P. (2013). *J. Synchrotron Rad.* **20**, 567–580. 10.1107/S0909049513009436 PMC3943555 23765298

